# Circular RNA cFAM210A, degradable by HBx, inhibits HCC tumorigenesis by suppressing YBX1 transactivation

**DOI:** 10.1038/s12276-023-01108-8

**Published:** 2023-11-01

**Authors:** Jian Yu, Wen Li, Guo-jun Hou, Da-peng Sun, Yuan Yang, Sheng-xian Yuan, Zhi-hui Dai, Hao-zan Yin, Shu-han Sun, Gang Huang, Wei-ping Zhou, Fu Yang

**Affiliations:** 1 The Department of General Surgery, Eastern Hepatobiliary Surgery Hospital, Naval Medical University, Shanghai, China; 2 The Third Department of Hepatic Surgery, Eastern Hepatobiliary Surgery Hospital, Naval Medical University, Shanghai, China; 3 The Department of Medical Genetics, Naval Medical University, Shanghai, China; 4 Shanghai Key Laboratory of Medical Bioprotection, Shanghai, 200433 China; 5 Key Laboratory of Biological Defense, Ministry of Education, Shanghai, 200433 China

**Keywords:** Liver cancer, Oncogenes

## Abstract

Hepatitis B protein x (HBx) has been reported to promote tumorigenesis in hepatitis B virus (HBV)-related hepatocellular carcinoma (HCC), but the mechanism awaits further investigation. In this study, we found that cFAM210A (a circular RNA derived from the third exon of transcript NM_001098801 of the FAM210A gene; CircBase ID: hsa_circ_0003979) can be silenced by HBx. cFAM210A expression was downregulated and negatively correlated with tumorigenesis in patients with HBV-related HCC. Furthermore, cFAM210A reduced the proliferation, stemness, and tumorigenicity of HCC cells. Mechanistically, HBx increased the N6-methyladenosine (m6A) level of cFAM210A by promoting the expression of RBM15 (an m6A methyltransferase), thus inducing the degradation of cFAM210A via the YTHDF2-HRSP12-RNase P/MRP pathway. cFAM210A bound to YBX1 and inhibited its phosphorylation, suppressing its transactivation function toward MET. These findings suggest the important role of circular RNAs in HBx-induced hepatocarcinogenesis and identify cFAM210A a potential target in the prevention and treatment of HBV-related HCC.

## Circular RNA cFAM210A: a new hope in HBV-related HCC treatment

A study has identified a circular RNA, cFAM210A, that inhibits the growth and stemness of hepatocellular carcinoma (HCC) cells. Researchers found that the hepatitis B virus (HBV) protein HBx promotes the degradation of cFAM210A through a process called N6-methyladenosine modification. This leads to increased tumour growth and stemness in HCC cells. By inhibiting the transactivation function of a protein called YBX1 on another protein called MET, cFAM210A suppresses tumour progression. These findings highlight the potential of cFAM210A as a target for the prevention and treatment of HBV-related HCC.

## Introduction

Hepatocellular carcinoma (HCC) is one of the most common and lethal cancers worldwide, and hepatitis B virus (HBV) infection is the main factor leading to HCC, especially in China^1^. Hepatitis B protein x (HBx) is essential in promoting tumorigenesis in HBV-related HCC^2,3^, but the underlying mechanisms have not been fully elucidated.

Circular RNAs (circRNAs), a specific series of noncoding RNAs with covalently closed, single-stranded structures and lacking a 5’ cap and 3’ poly(A) tail^4^, have long been considered “waste” produced during RNA splicing. In recent years, a large number of circRNAs in mammalian cells have been identified through high-throughput sequencing approaches^5^. Numerous studies have shed light on the important roles of circRNAs in the formation and development of almost all tumours, including those of gastric cancer^6^, breast cancer^7^, glioma^8^ and colorectal cancer^9^. In previous studies, we found that circRNA-cSMARCA5 can inhibit the growth and metastasis of HCC by sponging microRNAs^10^, and a plasma circRNA panel (containing three circRNAs) could be used to diagnose HBV-related HCC more accurately than the alpha-fetoprotein level^11^. However, whether HBx can promote tumorigenesis in HCC through circRNAs remains largely unknown.

In this study, we sequenced and characterized circRNAs in HBx-overexpressing (HBx-oe) and negative control (NC) HCC cells. Herein, we identified a set of differentially expressed circRNAs, among which cFAM210A (a circular RNA derived from the third exon of transcript NM_001098801 of the FAM210A gene; hg19, chr18:13681604-13682104; circBase ID: hsa_circ_0003979) exhibited the most notable decrease in expression. In addition, cFAM210A downregulation was confirmed to facilitate HBV-related hepatocarcinogenesis in clinical samples and promote stemness and tumorigenicity in HCC cells. Mechanistically, we revealed that HBx can increase N6-methyladenosine (m6A) modification of cFAM210A and facilitate its recognition and degradation. Using circRNA pull-down and RNA immunoprecipitation (RIP) assays, we discovered that cFAM210A can bind to the YBX1 protein, thus inhibiting its transactivation function.

## Methods

### Patients and samples

In total, 20 healthy liver tissues (distal healthy liver tissues from patients with hemangioma of the liver; Cohort 1) and 100 pairs of HCC and corresponding ANL tissues (divided into Cohorts 2 and 3) were obtained via surgical resection from patients with no preoperative treatment at Eastern Hepatobiliary Surgery Hospital (Shanghai, China). Human specimen collection was approved by the Ethics Committee of Eastern Hepatobiliary Surgery Hospital. Written informed consent forms were signed by each patient according to the policies of the committee. The samples in Cohorts 1, 2 and 3 were obtained in 2020, 2020, and 2015–2016, respectively. The detailed clinicopathological features are described in Supplementary Table 5.

### CircRNA pull-down assay

The circRNA pull-down assay was performed as previously described^12^. Briefly, the vector overexpressing MS2-fused cFAM210A (cFAM210A-MS2) and the vector overexpressing a Flag-tagged capture protein binding to MS2 (FCPM) were cotransfected into HepG2 cells. Then, the FCPM-cFAM210A-MS2-CAP (candidate protein) complexes were enriched by immunoprecipitation with an anti-Flag antibody (Fig. 7a, b, Group 5). The vectors without the MS2 or FCPM sequence were used as controls (Fig. 7b, Groups 6–8).

### Animal studies

The animal experiments in this study conformed to the Animal Research: Reporting of In Vivo Experiments (ARRIVE) guidelines (http://www.nc3rs.org.uk/arrive-guidelines) and were approved by the Ethics Committee of Eastern Hepatobiliary Surgery Hospital (Shanghai, China). The BALB/c nude mice used in this study were all male, 5 weeks old and purchased from Laboratory Animal Resources, Chinese Academy of Sciences (Beijing, China). All mice were housed in laminar flow cabinets under specific pathogen-free conditions at room temperature on a 12 h light/dark cycle, with food and water available ad libitum. The details of the in vivo extreme limiting dilution assay are described in Supplementary Data 2.

### Statistical analysis

All statistical analyses were performed using SPSS version 27.0 software (SPSS, Inc., Chicago, IL). For comparisons, the chi-square test, Student’s *t* test, the Wilcoxon signed-rank test or the Mann‒Whitney *U* test was used, as appropriate. Correlations were evaluated by Pearson correlation analysis. Survival curves were generated using the Kaplan‒Meier method, and differences in survival were assessed by the log-rank test. The estimated stem cell frequency in the extreme limiting dilution assays was calculated with ELDA (http://bioinf.wehi.edu.au/software/elda/). Statistical significance was indicated by *P* values of less than 0.05.

The remaining methods are described in Supplementary Data 2.

## Results

### cFAM210A is the main target of HBx

**Fig. 1:**
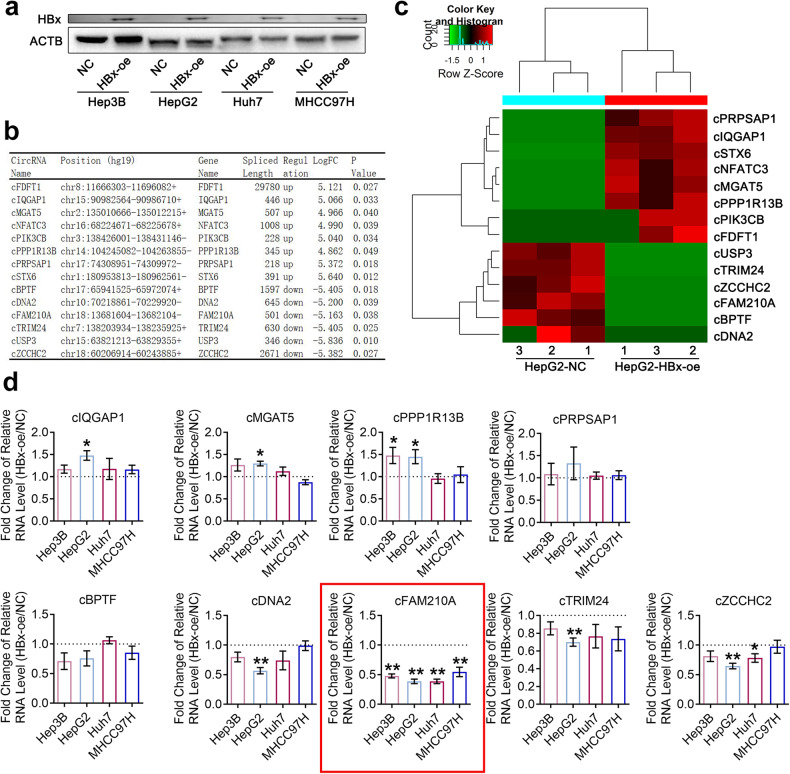
Screening and identification of HBx-regulated circRNAs. **a** Western blot analysis to measure the expression of HBx protein in HCC cell lines. ACTB was used as an endogenous control. **b** CircRNA-seq results showing the differentially expressed circRNAs between HBx-oe and NC HepG2 cells. **c** Cluster heatmap of differentially expressed molecules in the HBx-oe and NC groups. The horizontal axis shows the names of the circRNAs, and the vertical axis shows the NC and HBx-oe cell samples (3 replicates). **d** qRT‒PCR results validating the expression differences in nine circRNAs in four HCC cell lines. ACTB was used as an endogenous control. Student’s *t* test was used. NC negative control, HBx-oe HBx-overexpressing. *P < 0.05; **P < 0.01; ***P < 0.001.

First, the HBx protein was overexpressed in four human HCC cell lines (Hep3B, HepG2, Huh7 and MHCC97H) using lentivirus expressing HBx (Fig. 1a), which has been confirmed to promote HCC^13^. We then performed whole-transcriptome sequencing and identified differentially expressed circRNAs in HBx-overexpressing (HBx-oe) and NC (negative control) HepG2 cells. Among the 14 differentially expressed circRNAs after induction of HBx expression, 8 were upregulated, while 6 were downregulated (Supplementary Table 1 and Fig. 1b, c). By quantitative reverse transcription PCR (qRT‒PCR) using circRNA-specific divergent primers, agarose gel electrophoresis, Sanger sequencing and RNase R treatment assays^10^, we successfully validated the existence of 4 of the upregulated circRNAs (cIQGAP1, cMGAT5, cPPP1R13B and cPRPSAP1) and 5 of the downregulated circRNAs (cBPTF, cDNA2, cFAM210A, cTRIM24 and cZCCHC2) in HepG2 cells (Supplementary Fig. 1). Next, we measured the expression of these 9 circRNAs in HBx-oe and NC HCC cell lines. The expression of cFAM210A was the most obviously changed in all four HCC cell lines (Fig. 1d).

Therefore, we assumed that cFAM210A was the main target of HBx.

### cFAM210A expression is downregulated and negatively correlated with tumorigenesis in patients with HBV-related HCC

**Fig. 2:**
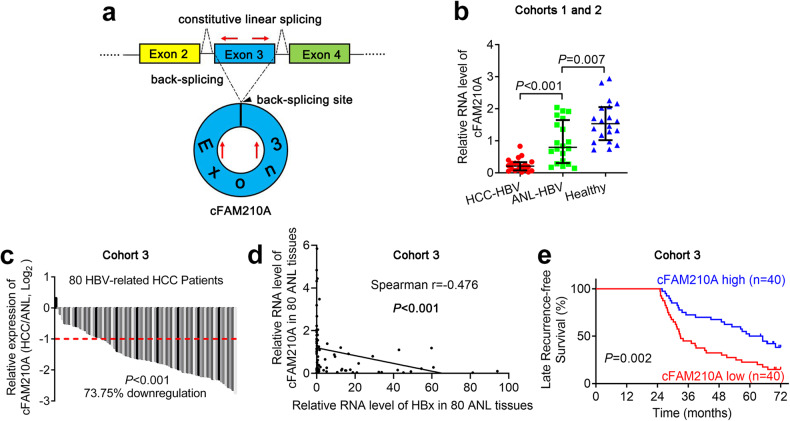
Clinical significance of cFAM210A expression. **a** Diagram of the pattern of cFAM210A biogenesis, with red arrows representing specific primers. **b** qRT‒PCR results showing the expression of cFAM210A in 20 healthy liver tissues (Cohort 1) and 20 paired HBV-related HCC and ANL tissues (Cohort 2). For comparisons between HCC-HBV and ANL-HBV samples, the Wilcoxon signed-rank test was used. For comparisons between ANL-HBV and healthy control samples, the Mann‒Whitney *U* test was used. **c** qRT‒PCR results comparing cFAM210A expression in HBV-associated HCC samples and non-HBV-related HCC samples. The Wilcoxon signed-rank test was used. **d** The correlation between the expression of HBx and that of cFAM210A in 80 ANL tissues was analyzed. **e** Kaplan‒Meier curve indicating the correlation between cFAM210A expression and cumulative late recurrence-free survival. The log-rank test was used. For (**b**), (**c**) and (**d**), ACTB was used as an endogenous control.

cFAM210A is derived from the third exon of the NM_001098801 transcript of the FAM210A gene by back-splicing (Fig. 2a), and its length is 501 nucleotides. We then measured the expression of cFAM210A in 20 healthy liver tissues (distal healthy liver tissues from patients with cavernous hemangioma of the liver; Cohort 1) and 20 paired HBV-related HCC and adjacent noncancerous liver (ANL) tissues (Cohort 2). The expression of cFAM210A in HCC tissues was lower than that in ANL tissues, indicating an antitumor role of cFAM210A (Fig. 2b). Importantly, the expression of cFAM210A in HBV-related ANL tissues was lower than that in healthy tissues, indicating that cFAM210A may be downregulated by HBV infection (Fig. 2b).

There are two types of HCC recurrence after hepatectomy: early recurrence (within 2 years after surgery), which arises from residual HCC cells disseminated in the remnant liver; and late recurrence (more than two years after surgery), constituting de novo tumorigenesis independent of the completely resected primary tumor^14^. Gene expression analysis of ANL tissue can predict the risk of late recurrence of HCC following hepatectomy^14^. To determine whether cFAM210A is associated with de novo tumorigenesis of HCC, we collected HCC and ANL tissues as well as prognostic data from 80 patients with HBV-related HCC with late recurrence following surgery (Cohort 3). qRT‒PCR analysis of the tissues in Cohort 3 confirmed the downregulation of cFAM210A in HCC (Fig. 2c) and showed that the expression of cFAM210A was negatively correlated with the level of HBx in ANL tissues (Fig. 2d, Spearman r = −0.476, *P* < 0.001). Based on the median cFAM210A expression level in ANL tissues, we divided Cohort 3 into the cFAM210A-high group (*n* = 40) and the cFAM210A-low group (*n* = 40). Kaplan‒Meier survival analysis showed that the cFAM210A-low group had poorer late recurrence-free survival (*P* = 0.002) (Fig. 2e).

Taken together, these results showed that cFAM210A expression was negatively correlated with tumorigenesis in patients with HBV-related HCC.

### HBx promotes the degradation of cFAM210A by increasing its m6A level

The abundance of a circRNA is determined by its biogenesis and degradation. CircRNAs are produced from precursor mRNAs (pre-mRNAs) by back-splicing, which can be affected by cis-acting elements (long flanking introns and intronic complementary sequences) and trans-acting factors (ADAR1, DHX9, FUS, NF90/NF110 and QKI)^5,15^. On the other hand, circRNAs can be degraded in four ways^15^: (i) circRNAs can be degraded by RNase L upon cellular infection with a double-stranded RNA (dsRNA) virus^16^; (ii) highly structured circRNAs can be undergo decay mediated by UPF1 and G3BP1^17^; (iii) circRNAs can be degraded after being bound by microRNAs in an AGO2-dependent manner in unique situations^18^; and (iv) m6A-modified circRNAs can be degraded through an endoribonucleolytic cleavage pathway, that is, the YTHDF2-HRSP12-RNase P/MRP pathway^19^. In this pathway, YTHDF2 (an m6A reader), HRSP12 (an adaptor that bridges YTHDF2 and RNase P/MRP) and POP1 (a component of the endoribonuclease RNase P/MRP) are key proteins^19^.

First, we explored whether HBx can affect the biogenesis of cFAM210A. qRT‒PCR and Western blot analyses showed that the level of the FAM210A pre-mRNA (pFAM210A) and the mRNA and protein levels of FAM210A and the tested trans-acting factors did not show significant changes after HBx overexpression (Supplementary Fig. 2), indicating that HBx may not affect the transcription of either the FAM210A gene or the trans-acting factors of cFAM210A. In addition, the HBx protein could not directly affect the cis-acting elements of cFAM210A due to its inability to bind to nucleic acids independently^20^. Therefore, we assumed that HBx could not affect the biogenesis of cFAM210A.

**Fig. 3:**
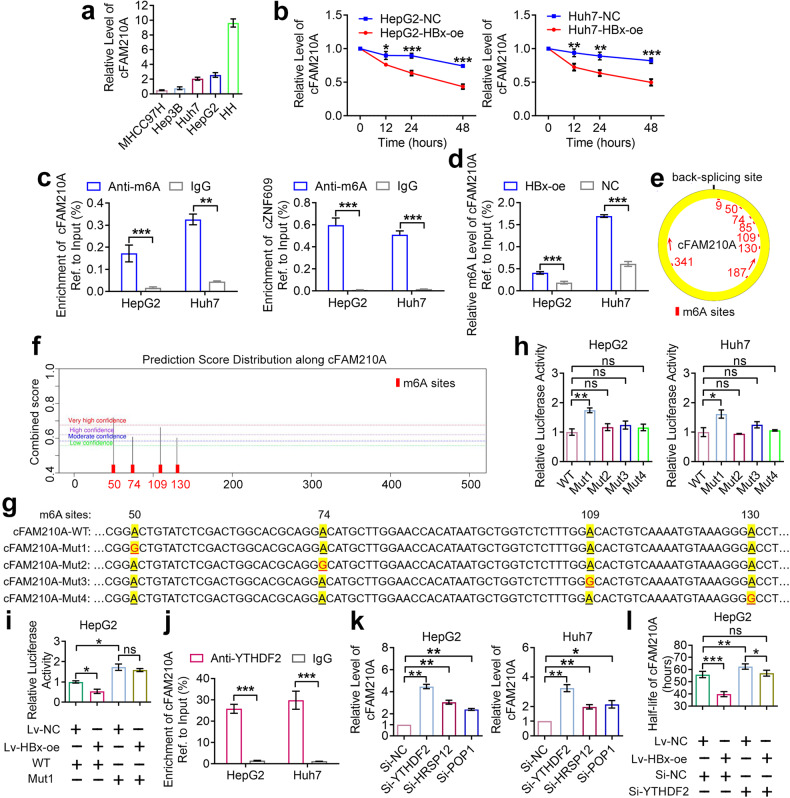
HBx promotes the degradation of cFAM210A by increasing its m6A level. **a** qRT‒PCR results showing the expression of cFAM210A in human hepatocytes (HHs) and four HCC cell lines. **b** qRT‒PCR results showing the expression of cFAM210A after treatment with actinomycin D at the indicated time points. **c** MeRIP-qPCR analysis of m6A-modified cFAM210A in HCC cell lines. cZNF609 was used as a positive control. **d** MeRIP-qPCR analysis of m6A-modified cFAM210A in HBx-oe and NC cell lines. **e**, **f** The m6A sites of cFAM210A were predicted by CircPrimer 2.0 software (**e**) and SRAMP (**f**). The red arrows represent the primers used for MeRIP-qPCR. **g** Schematic representation of mutated m6A sites in cFAM210A. **h** Dual-luciferase assay result showing the relative luciferase activity of the wild-type (WT) and mutant (Mut)1/2/3/4 reporters. **i** Rescue assay results demonstrating that the relative luciferase activity of the WT reporter but not the Mut1 reporter was decreased by overexpression of HBx. **j** RIP-qPCR using an anti-YTHDF2 antibody was performed in HCC cell lines. **k** qRT‒PCR results showing the expression of cFAM210A after knockdown of YTHDF2, HRSP12 or POP1. **l** The actinomycin D treatment assay demonstrated that the half-life of cFAM210A was rescued by HBx overexpression in YTHDF2-depleted HepG2 cells. Student’s *t* test was used. NC negative control, HBx-oe HBx-overexpressing, ns not significant. *P < 0.05; **P < 0.01; ***P < 0.001. For (**a**) and (**k**), ACTB was used as an endogenous control.

Then, we explored whether HBx can affect the degradation of cFAM210A. qRT‒PCR analysis showed that the expression of cFAM210A in primary human hepatocytes (HHs) was higher than that in HCC cells (HepG2, Huh7, Hep3B and MHCC97H), while its expression in HepG2 and Huh7 cells was higher than that in Hep3B and MHCC97H cells (Fig. 3a). After treatment with actinomycin D to block the biogenesis of cFAM210A, HBx overexpression resulted in significant downregulation of cFAM210A expression (Fig. 3b), indicating that HBx could promote the degradation of cFAM210A. As HBV is not a dsRNA virus, cFAM210A is not a highly structured circRNA (defined as a circRNA with a −ΔG/nt value ≥ 0.300; the −ΔG/nt value of cFAM210A was 0.254)^17^, and cFAM210A could not bind to AGO2 (Supplementary Fig. 3a); thus, the first three mechanisms of degradation are not applicable to cFAM210A.

Subsequently, we tried to investigate whether cFAM210A can be degraded by HBx in an m6A-dependent manner. The results of methylated RNA immunoprecipitation (MeRIP)-qPCR showed that cFAM210A was m6A modified (Fig. 3c; cZNF609, a well-known^21,22^ circular RNA, was used as a positive control) and that its relative m6A level was increased after HBx overexpression (Fig. 3d). Furthermore, four m6A sites (50, 74, 109 and 130) were predicted both by CircPrimer 2.0^23^ (Fig. 3e) and SRAMP^24^ (Fig. 3f). As a dual-luciferase assay can be used to determine the key m6A sites^25^, we generated dual-luciferase reporter vectors by inserting the sequence of wild-type (WT) cFAM210A or mutant (Mut)1/2/3/4 (A to G; Mut1: site 50, Mut2: site 74; Mut3: site 109; Mut4: site 130) cFAM210A (Fig. 3g). The relative luciferase activity of the Mut1 (but not the Mut2, Mut3 or Mut4) reporter was higher than that of the WT reporter in both HepG2 and Huh7 cells (Fig. 3h), indicating that m6A site 50 is the key m6A site in cFAM210A. Moreover, the relative luciferase activity of the WT reporter but not the Mut1 reporter was decreased by overexpression of HBx (Fig. 3i). Therefore, we concluded that HBx could promote cFAM210A degradation by increasing its m6A level at m6A site 50.

Importantly, cFAM210A could be enriched by YTHDF2 (Fig. 3j) and was significantly upregulated upon knockdown of YTHDF2, HRSP12 or POP1 (Fig. 3k, Supplementary Fig. 3b). Furthermore, the half-life of cFAM210A was significantly increased after knockdown of YTHDF2 in HepG2 cells, and this increase was reversed by HBx overexpression (Fig. 3l, Supplementary Fig. 3c). Finally, HBx did not affect the expression of YTHDF2, HRSP12 or POP1 (Supplementary Fig. 3d, e).

In addition, the results of m6A dot blot assays showed that HBx increased the global m6A level of RNA extracted from HCC cells (Supplementary Fig. 4).

In summary, HBx can promote the m6A modification of cFAM210A at m6A site 50, and m6A-modified cFAM210A can be degraded via the YTHDF2-HRSP12-RNase P/MRP pathway.

### HBx increases the m6A level of cFAM210A by promoting the expression of RBM15

**Fig. 4:**
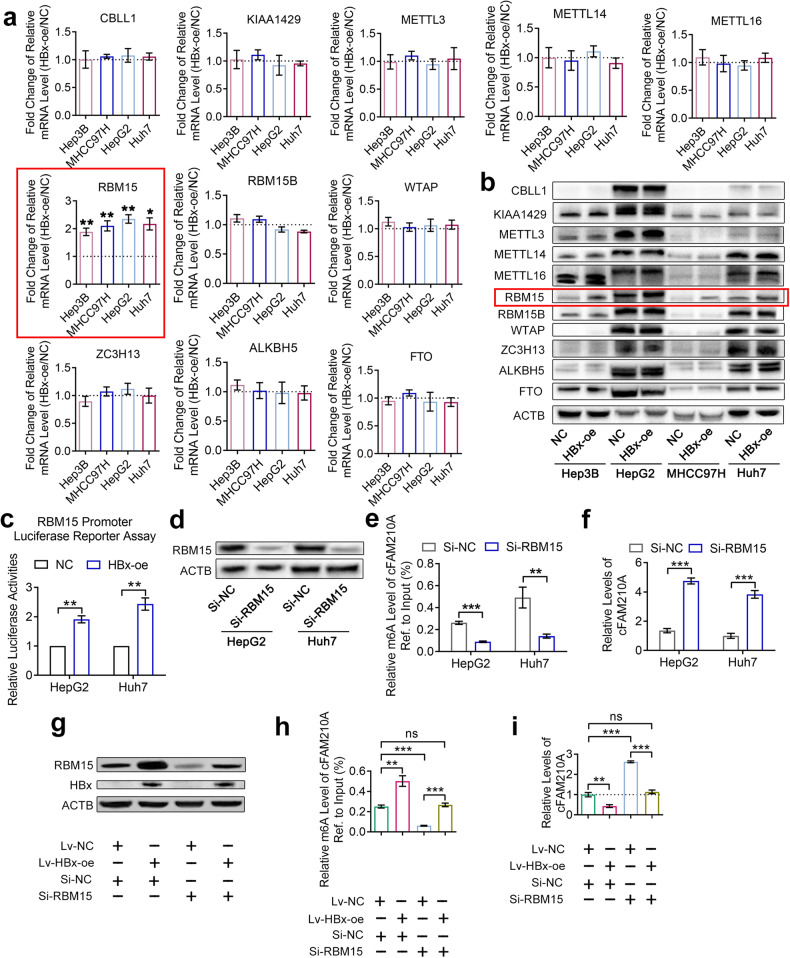
HBx increases the m6A level of cFAM210A by promoting the expression of RBM15. **a, b** qRT‒PCR (**a**) and Western blot (**b**) results showing the mRNA and protein levels of m6A regulators in HBx-oe and NC cells. **c** Dual-luciferase assay results indicating the activity of the RBM15 gene promoter in HBx-oe and NC cells. **d** Western blot results showing the knockdown efficiency of si-RBM15 in HCC cells. **e**, **f** MeRIP-qPCR (**e**) and qRT‒PCR (**f**) results showing the m6A level and expression of cFAM210A in si-RBM15 and si-NC cells. **g** Western blot results showing the knockdown efficiency of si-RBM15 and overexpression efficiency of the HBx-oe vector in HepG2 cells. **h**, **i** MeRIP-qPCR (**h**) and qRT‒PCR (**i**) results in HepG2 cells showing that the effects of si-RBM15 were abolished by overexpressing HBx. For (**a**), (**f**) and (**i**), ACTB was used as an endogenous control. For (**a**), (**c**), (**e**), (**f**), (**h**) and (**i**), Student’s *t* test was used. NC negative control, HBx-oe HBx-overexpressing, ns not significant. *P < 0.05; **P < 0.01; ***P < 0.001.

It has been reported that m6A methyltransferases (CBLL1 (HAKAI), KIAA1429 (VIRMA), METTL3, METTL14, METTL16 (METT10D), RBM15, RBM15B, WTAP and ZC3H13) can increase the m6A level of RNA, while m6A demethylases (ALKBH5 and FTO) can decrease the m6A level of RNA^26^. qRT‒PCR (Fig. 4a) and Western blot (Fig. 4b) analyses demonstrated that RBM15 but not the other methyltransferases or demethylases was significantly upregulated after HBx overexpression in HCC cell lines. In the dual-luciferase assay with a reporter containing the RBM15 promoter, overexpression of HBx promoted RBM15 transcription (Fig. 4c). Therefore, we concluded that HBx promoted the expression of RBM15 by inducing its transcription.

After knocking down RBM15 (Fig. 4d), both the m6A level (Fig. 4e) and the expression (Fig. 4f) of cFAM210A were significantly decreased. Importantly, this effect was abolished by overexpressing HBx simultaneously (Fig. 4g–i).

In summary, HBx can increase the m6A level of cFAM210A by promoting the expression of RBM15 through transcriptional induction.

### cFAM210A inhibits the proliferation, stemness, and tumorigenicity of HCC cells

**Fig. 5:**
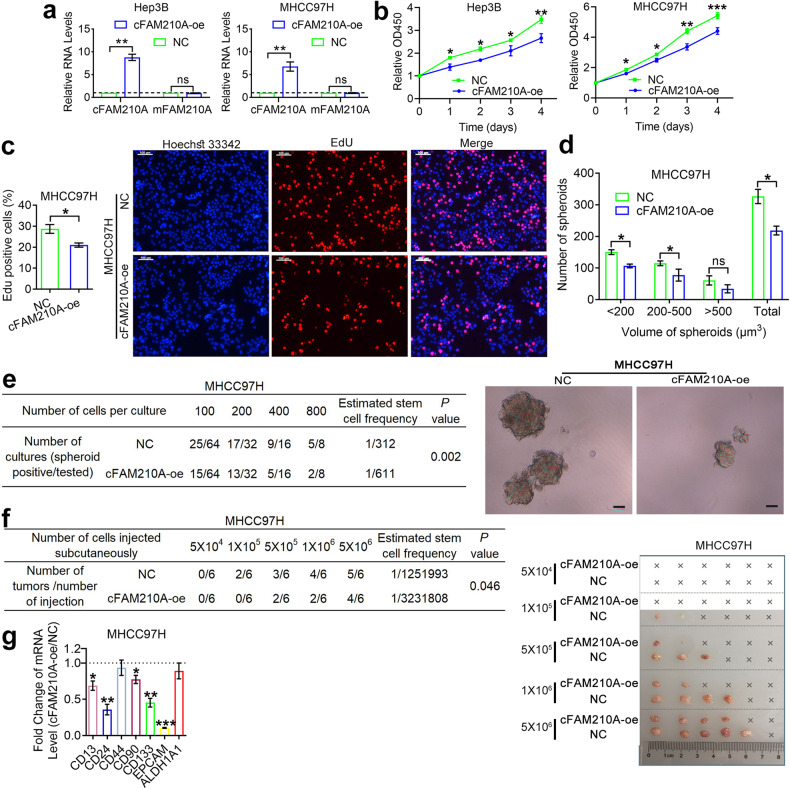
Overexpressing cFAM210A inhibits the proliferation, stemness, and tumorigenicity of HCC cells. **a** qPCR results showing the expression of cFAM210A and mFAM210A after infection with cFAM210A-oe lentivirus and its negative control. **b** CCK-8 assays were performed in cFAM210A-oe HCC cells and their negative controls. **c** EdU incorporation assays were performed in MHCC97H cells (left). Representative images; scale bar, 100 μm (right). **d** Spheroid formation assays were performed in MHCC97H cells (top). Representative images (bottom). **e**, **f** In vitro (**e**) and in vivo (**f**) limiting dilution assays. The estimated stem cell frequency of each group was calculated by ELDA (http://bioinf.wehi.edu.au/software/elda/). **g** qRT‒PCR showing the expression of seven markers of cancer stem cells in HCC. For (**a**) and (**g**), ACTB was used as an endogenous control. For (**a**–**d**) and (**g**), Student’s *t* test was used. NC negative control, cFAM210A-oe cFAM210A-overexpressing lentivirus, ns not significant. *P < 0.05; **P < 0.01; ***P < 0.001.

To explore the biological functions of cFAM210A in HCC, we overexpressed cFAM210A in Hep3B and MHCC97H cells using a lentiviral vector (Fig. 5a). Notably, the mRNA level of FAM210A (mFAM210A) did not show significant changes after cFAM210A overexpression (Fig. 5a). The CCK-8 (Fig. 5b) and EdU incorporation (Fig. 5c) assay results showed that the proliferation ability of HCC cells was significantly reduced after cFAM210A overexpression. The results of spheroid formation assays (Fig. 5d) and in vitro (Fig. 5e) and in vivo (Fig. 5f) limiting dilution assays demonstrated that forced expression of cFAM210A suppressed the stemness and tumorigenicity of HCC cells. Furthermore, among the seven markers (CD13, CD24, CD44, CD90, CD133, EPCAM and ALDH1A1)^27^ of cancer stem cells (CSCs) in HCC, five were downregulated after cFAM210A overexpression (Fig. 5g).

**Fig. 6:**
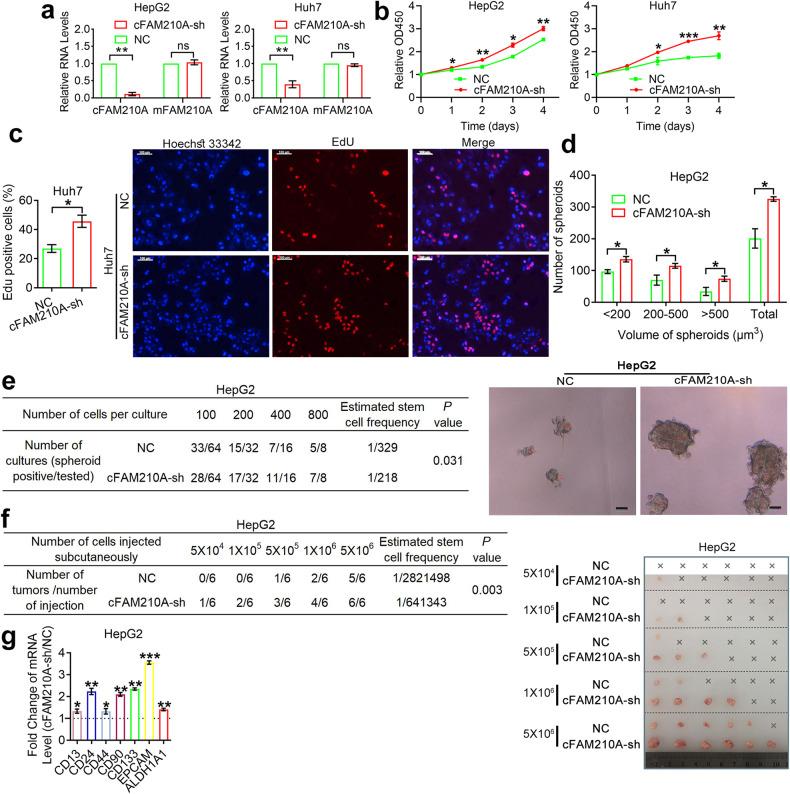
Knocking down cFAM210A promotes the proliferation, stemness, and tumorigenicity of HCC cells. **a** qPCR results showing the expression of cFAM210A and mFAM210A after using cFAM210A-sh lentivirus and its negative control. **b** CCK-8 assays were performed in cFAM210A-sh HCC cells and their negative controls. **c** EdU incorporation assays were performed in Huh7 cells (left). Representative images; scale bar, 100 μm (right). **d** Spheroid formation assays were performed in HepG2 cells (top). Representative images (bottom). **e**, **f** In vitro (**e**) and in vivo (**f**) limiting dilution assays. The estimated stem cell frequency of each group was calculated by ELDA (http://bioinf.wehi.edu.au/software/elda/); scale bar, 50 μm. **g** qRT‒PCR showing the expression of seven markers of cancer stem cells in HCC. For (**a**) and (**g**), ACTB was used as an endogenous control. For (**a**–**d**) and (**g**), Student’s *t* test was used. NC negative control, cFAM210A-sh, lentivirus-mediated short hairpin RNA against cFAM210A. ns not significant. *P < 0.05; **P < 0.01; ***P < 0.001.

Next, we successfully knocked down cFAM210A in HepG2 and Huh7 cells (Fig. 6a). Consistent with the above findings, the CCK-8 (Fig. 6b) and EdU incorporation (Fig. 6c) assay results indicated the proliferation-inhibiting ability of cFAM210A. The results of spheroid formation assays (Fig. 6d) and in vitro (Fig. 6e) and in vivo (Fig. 6f) limiting dilution assays showed that the stemness and tumorigenicity of HCC cells were enhanced by knockdown of cFAM210A. Moreover, all seven detected markers of CSCs in HCC were upregulated after silencing of cFAM210A (Fig. 6g).

Collectively, these findings indicated that cFAM210A inhibited the proliferation, stemness, and tumorigenicity of HCC cells in vitro and in vivo.

### cFAM210A can bind to YBX1

It has been reported that circRNAs can function in the following three ways^15^: (i) binding to microRNAs in an AGO2-dependent manner^5^; (ii) being translated into proteins or peptides; and (iii) binding to proteins.

First, the results of RIP-qPCR using an anti-AGO2 antibody showed that cFAM210A could not bind to AGO2 (Supplementary Fig. 3a), meaning that cFAM210A may not bind to microRNAs. Furthermore, although circRNADb (http://reprod.njmu.edu.cn/cgi-bin/circrnadb/circRNADb.php) predicted that cFAM210A could be translated into a 159 AA protein, this protein could not be detected by Western blotting (Supplementary Fig. 5). In addition, SProtP Human (http://reprod.njmu.edu.cn/cgi-bin/sprotp/api.py) indicated that the estimated half-life of the predicted cFAM210A protein, even if it did exist, was very short (<30 min). Therefore, we concluded that cFAM210A may not function through translation into a protein or peptide.

**Fig. 7:**
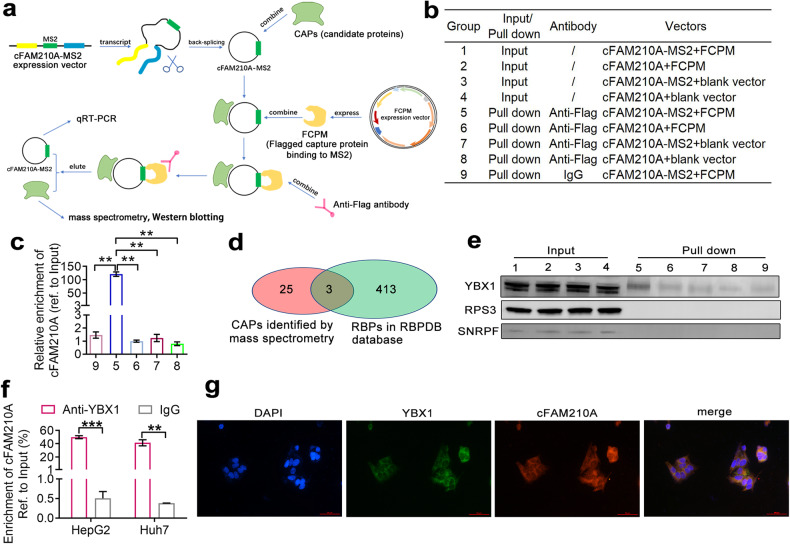
cFAM210A can bind to YBX1. **a** Schematic of the circRNA pull-down assay. **b** Grouping scheme used for circRNA pull-down assays. **c** qRT‒PCR following circRNA pull-down showed the enrichment of cFAM210A in Group 5. **d** Screening of proteins binding to cFAM210A. **e** Western blot results validating the candidate proteins binding to cFAM210A. **f** RIP-qPCR using an anti-YBX1 antibody in HCC cell lines. **g** Dual RNA fluorescence in situ hybridization (FISH) and immunofluorescence assay results showing the colocalization of cFAM210A and YBX1 in HepG2 cells. Scale bar, 100 μm. CAPs candidate proteins, FCPM flag-tagged capture protein binding to MS2. For (**e**), Student’s *t* test was used. ns not significant. *P < 0.05; **P < 0.01; ***P < 0.001.

To explore whether cFAM210A can bind to proteins (candidate proteins, CAPs), we performed a circRNA pull-down assay as previously described^12^. Briefly, a vector overexpressing MS2-fused cFAM210A (cFAM210A-MS2) and a vector overexpressing a Flag-tagged capture protein binding to MS2 (FCPM) were cotransfected into HepG2 cells. Then, the FCPM-cFAM210A-MS2-CAP complexes were enriched by immunoprecipitation with an anti-Flag antibody (Fig. 7a, b, Group 5). The vectors without the MS2 or FCPM sequence were used as controls (Fig. 7b, Groups 6–8). Subsequent Western blotting using an anti-Flag antibody showed that FCPM was successfully pulled down in Groups 5 and 6 (Supplementary Fig. 6a, b), while qRT‒PCR analysis indicated that cFAM210A was successfully pulled down only in Group 5 (Fig. 7c). The enriched complexes in Groups 5 and 6 were then analyzed by mass spectrometry. We detected 77 proteins in Group 5 (Supplementary Table 2) and 138 proteins in Group 6 (Supplementary Table 3). Among the detected proteins, 28 (Supplementary Table 4) were detected only in Group 5 (but not in Group 6) and were thought to be candidate proteins binding to cFAM210A.

We then overlapped these 28 candidate proteins with 416 established RNA binding proteins (RBPs) (http://rbpdb.ccbr.utoronto.ca)^28,29^ and obtained three candidate proteins: YBX1, RPS3 and SNRPF (Fig. 7d). CircRNA pull-down followed by western blotting (Fig. 7e), RIP-qPCR using antibodies against these three proteins (Fig. 7f, Supplementary Fig. 6c) and dual RNA fluorescence in situ hybridization (FISH) and immunofluorescence staining (Fig. 7g) demonstrated that cFAM210A bound to YBX1 but not PRS3 or SNRPF.

Moreover, RIP-qPCR using an anti-YBX1 antibody showed that mFAM210A cannot bind to YBX1 (Supplementary Fig. 7a). Compared to the sequence of mFAM210A, the sequence of cFAM210A differs only in the region spanning its back-splice site (BSS). Therefore, we assumed that the sequence spanning the BSS of cFAM210A was the key sequence for its binding to YBX1. To test this hypothesis, the vector overexpressing MS2-fused cFAM210A and lacking the 50 nucleotides spanning the BSS (cFAM210A-MS2-△BSS50) (Supplementary Fig. 7b) and the FCPM vector were cotransfected into HepG2 cells. Subsequent circRNA pull-down followed by Western blotting showed that YBX1 could not be pulled down (Supplementary Fig. 7c). These experiments demonstrated that the key YBX1-binding sequence in cFAM210A was located in the 50 nucleotides spanning its BSS.

In summary, cFAM210A can function by binding to the YBX1 protein but not by other mechanisms.

### cFAM210A inhibits the phosphorylation of YBX1, suppressing its transactivation function toward MET

CircRNAs can bind to proteins, resulting in their translocation (from the nucleus to the cytoplasm, or vice versa)^30^ or affecting their functions^30^ or stability^31,32^. YBX1 can interact directly with promoters and induce the transcription of various genes^33–36^. Among these genes, CD44^37^, MET^37^ and MDR1^38^ have been reported to play important roles in HCC stemness.

**Fig. 8:**
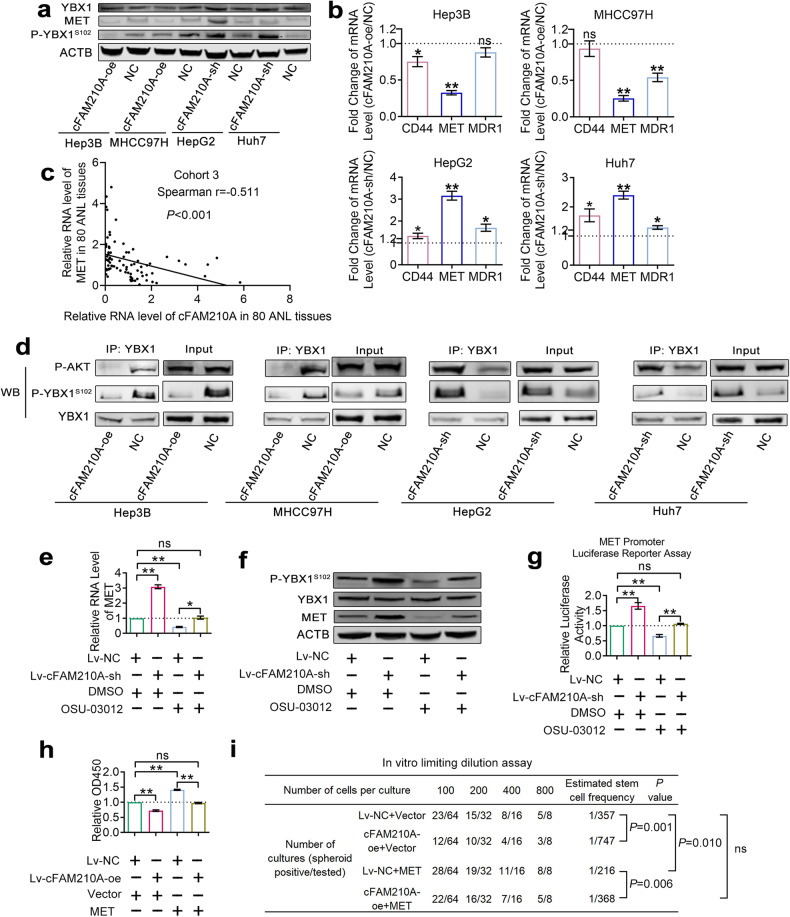
cFAM210A inhibits the phosphorylation of YBX1, suppressing its transactivation function toward MET. **a** Western blot results showing the protein levels of MET, YBX1 and P-YBX1^S102^ upon overexpression or silencing of cFAM210A. **b** qRT‒PCR results showing the expression of CD44, MET and MDR1 upon overexpression or silencing of cFAM210A. **c** The correlation between the RNA level of MET and that of cFAM210A in 80 ANL tissues was analyzed. **d** Western blot analysis following immunoprecipitation (IP) after overexpression or silencing of cFAM210A. **e**–**g** qRT‒PCR (**e**), Western blot (**f**) and MET promoter luciferase reporter assay (**g**) results showing that OSU-03012 suppressed the phosphorylation of YBX1^S102^ and downregulated the expression of MET, while these effects were abolished by silencing cFAM210A. **h**–**i** The CCK-8 assay (OD450 was measured on the third day) (**h**) and in vitro limiting dilution assay (**i**) results demonstrated that MET promoted the proliferation and stemness of HCC cells, while these effects were abolished by overexpressing cFAM210A. For (**b**), (**e**), (**g**) and (**h**), Student’s *t* test was used. For (**i**), the estimated stem cell frequency of each group was calculated by ELDA (http://bioinf.wehi.edu.au/software/elda/). NC negative control; cFAM210A-oe cFAM210A-overexpressing lentivirus, cFAM210A-sh lentivirus-mediated short hairpin RNA against cFAM210A, ns not significant. *P < 0.05; **P < 0.01; ***P < 0.001.

Western blotting showed that the expression (Fig. 8a) and localization (Supplementary Fig. 8) of YBX1 did not change significantly after overexpression or knockdown of cFAM210A. qRT‒PCR showed that cFAM210A inhibited the mRNA expression of YBX1-transactivated genes (CD44, MET and MDR1) (Fig. 8b). Among these genes, MET exhibited the most obvious change (Fig. 8b). Furthermore, the protein level of MET was decreased by cFAM210A (Fig. 8a). Importantly, the RNA levels of cFAM210A and MET were positively correlated in ANL tissues of patients with HBV-related HCC (Fig. 8c). Therefore, we concluded that cFAM210A could inhibit the ability of YBX1 to transactivate MET.

Then, we tried to explore the related mechanism. It has been reported that phosphoinositide dependent kinase-1 (PDK-1) can activate AKT by phosphorylating it and that phosphorylated AKT (P-AKT) can bind to and phosphorylate YBX1 at Ser102 (YBX1^S102^)^39,40^. YBX1 can bind to the promoter of MET, and phosphorylation of YBX1^S102^ could strengthen its transactivation function toward MET^35^. Furthermore, treatment with OSU-03012 (a PDK1 inhibitor) was found to decrease the phosphorylation of YBX1^S102,^^35,40^, thus inhibiting the transcription of MET, in normal and malignant human mammary cells^35^.

Interestingly, we found that cFAM210A inhibited the phosphorylation of YBX1^S102^ (Fig. 8a). Furthermore, immunoprecipitation (IP) experiments using an anti-YBX1 antibody showed that the enrichment of P-AKT decreased after overexpressing cFAM210A but increased after knockdown of cFAM210A (Fig. 8d). This result indicated that cFAM210A may prevent P-AKT from binding to YBX1. Moreover, qRT‒PCR analysis (Fig. 8e), Western blot analysis (Fig. 8f) and the MET promoter luciferase reporter assay (Fig. 8g) showed that OSU-03012 treatment suppressed the phosphorylation of YBX1^S102^ and downregulated the transcription and expression of MET, while these effects were abolished by silencing cFAM210A. Therefore, we concluded that cFAM210A suppressed the transcription of MET by inhibiting the phosphorylation of YBX1^S102^.

In addition, the results of CCK-8 (Fig. 8h) and in vitro limiting dilution (Fig. 8i) assays demonstrated that MET promoted the proliferation and stemness of HCC cells, while these effects were attenuated by overexpressing cFAM210A.

Finally, in HBV-infected HepG2.2.15^41,42^ and HepAD38^43,44^ cells, knockdown of HBx led to downregulation of RBM15 and MET, suppression of YBX1^S102^ phosphorylation and upregulation of cFAM210A (Supplementary Fig. 9a, b), while overexpression of HBx had the opposite effects (Supplementary Fig. 9b, c). These results further confirmed our findings.

In conclusion, cFAM210A, which is degraded in response to HBx-mediated m6A modification, inhibited tumorigenesis in HCC by suppressing the transactivation function of YBX1 toward MET. The graphical abstract of this study is shown in Supplementary Fig. 10.

## Discussion

FAM210A has been reported to increase bone and muscle strength^45^ and to influence cardiac remodelling during heart failure^46^, but the role of FAM210A in tumours has not been mentioned before. In this study, we found that a circRNA derived from the FAM210A gene inhibited tumorigenesis in HBV-related HCC, demonstrating the function of the FAM210A gene in another aspect.

The expression levels of circRNAs are determined by multiple factors, and the modes of action of circRNAs vary^15^. In the present research, by a process of elimination, we determined that the downregulation of cFAM210A after HBx overexpression was attributed to m6A-dependent degradation via the YTHDF2-HRSP12-RNase P/MRP pathway and that cFAM210A inhibited tumorigenesis in HCC by suppressing the transactivation function of YBX1 toward MET but not by other mechanisms. To the best of our knowledge, this is the first report on the function of cFAM210A.

Rao et al.^47^. found that HBx could upregulate the expression of METTL3. However, they investigated only one m6A methyltransferase (METTL3) in a single cell line (HepG2), and the mechanism was not explored. In the present study, we investigated all known m6A methyltransferases and m6A demethylases in four HCC cell lines and found that RBM15 but not METTL3 was upregulated in all four cell lines. Mechanistically, the dual-luciferase assay results showed that HBx promoted the transcription of RBM15. Consistent with this finding, transcriptome sequencing data from a public database (https://www.ncbi.nlm.nih.gov/geo/query/acc.cgi?acc=GSE64875)^48^ also suggested that the expression of RBM15 in HBx-oe HepG2 cells was higher than that in negative control cells (adjusted *P* = 0.021, fold change = 1.547).

A few studies^47,49^ have reported that m6A modification can affect the back-splicing of a subset of circRNAs, resulting in higher expression of these circRNAs and lower expression of the corresponding pre-mRNAs and mRNAs. However, in this research, after HBx overexpression, the m6A level of cFAM210A was increased, facilitating its degradation via the YTHDF2-HRSP12-RNase P/MRP pathway, while the expression of pFAM210A and mFAM210A did not show significant changes. These findings suggest the multifaceted roles of m6A modification of circRNAs^50^.

YBX1 is a well-known RBP with multiple functions^33^. YBX1 has been shown to be upregulated^51^, to be associated with poorer prognosis^52^, to promote tumorigenesis^52^ and to mediate sorafenib resistance^53^ in HCC. Several studies have reported that circRNAs can bind to YBX1 to affect its stability, location or function^30^. For example, circRNA-SORE can sequester YBX1 in the cytoplasm and prevent it from being degraded by nuclear PRP19^31^, while circNfix can mediate the degradation of Ybx1 by inducing its ubiquitination in mice^32^. CircAnks1a can recruit Ybx1 to the promoter of Vegfb and activate its transcription in rodents^54^. CircACTN4 can recruit YBX1 to the promoter of FZD7 and activate its transcription in human intrahepatic cholangiocarcinoma cells^55^. In this research, we first reported that circRNA-cFAM210A can inhibit the phosphorylation of YBX1 to suppress its transactivation function, uncovering another mode of action linking circRNAs and YBX1. Notably, YBX1 can function in various processes, including transactivation, DNA repair, DNA replication, pre-mRNA splicing, and disassembly of nucleoli^33^. However, whether cFAM210A can affect the functions of YBX1, except for transactivation, in other ways needs to be further explored.

In addition, the mechanism by which cFAM201A is involved in the phosphorylation of YBX1 awaits further investigation.

In summary, this study suggests the important role of circular RNAs in HBx-induced hepatocarcinogenesis, making cFAM210A a potential target in the prevention and treatment of HBV-related HCC.

### Supplementary information

Supplementary Data 1

Supplementary Data 2

## Data Availability

The Gene Expression Omnibus accession number for the whole-transcriptome sequencing data in HBx-overexpressing (HBx-oe) and NC (negative control) HepG2 cells is GSE215232.
