# Supplementary material for: Circular RNA cFAM210A, degradable by HBx, inhibits HCC tumorigenesis by suppressing YBX1 transactivation
Source: Exp Mol Med. 2023 Nov 1;55(11):2390–401. doi: 10.1038/s12276-023-01108-8 (PMC10689457; doi:10.1038/s12276-023-01108-8)
Supplement: Supplementary file 2 — Supplementary Data 2 [file 12276_2023_1108_MOESM2_ESM.pdf]

Circular RNA cFAM210A, degradable by HBx, inhibits HCC tumorigenesis by suppressing YBX1 transactivation

(Supplementary Tables. 1-4 are presented in Supplementary Data 1)

Table of contents

Supplementary materials and methods……………………………………………………2

Supplementary Table. 5...………………………………………………………………6

Supplementary Table. 6...………………………………………………………………7

Supplementary Table. 7...………………………………………………………………10

Supplementary Fig. 1…………………………………………………………………….11

Supplementary Fig. 2…………………………………………………………………….12

Supplementary Fig. 3……………………………………………………………………13

Supplementary Fig. 4……………………………………………………………………14

Supplementary Fig. 5……………………………………………………………………15

Supplementary Fig. 6…………………………………………………………………….16

Supplementary Fig. 7…………………………………………………………………….17

Supplementary Fig. 8……………………………………………………………………18

Supplementary Fig. 9……………………………………………………………………19

Supplementary Fig. 10…………………………………………………………………20

References ……………………………………………………………………… …21

Supplementary materials and methods

Follow-up

The patients of cohort 3 received check-ups every 2–3 months after surgery during the first 24 months and every 3–6 months thereafter until November 10, 2016. The median follow-up period was 45.2 months. Physicians who were blinded to the study performed the follow-up examinations. Serum AFP levels and abdominal ultrasound examinations were performed every month during the first year after surgery and every 3–6 months thereafter. Computed tomography and/or magnetic resonance imaging were performed every 3–6 months or when a recurrence was suspected. The diagnosis of recurrence was based on the diagnosis criteria from the AASLD Practice Guidelines (http://www.aasld.org/practiceguidelines/Documents/Bookmarked%20Practice%20Guidelines/HCCUpdate2010.pdf) Once recurrence was confirmed, further treatment was implemented based on the tumor diameter, the number of tumors, the location of the tumor, and the extent of vessel invasion as well as liver function and performance statuses. Recurrence-free survival (RFS) was calculated from the date of tumor resection until the detection of tumor recurrence, death from a cause other than HCC, or the last follow-up visit.

Whole transcriptome sequencing and identification of differentially expressed circRNAs

Total RNA was extracted from HBx-overexpressing (HBx-oe) and NC (negative control) HepG2 cells using Trizol reagent (Invitrogen, CA) following the manufacturer's protocol. The RNA concentration and quality were evaluated with the NanoDrop 2000 Spectrophotometer (Thermo Fisher Scientific, Wilmington, DE, USA) and Agilent 2100 Bioanalyzer (Agilent Technologies).

Firstly, rRNA was depleted by NEBNext rRNA Depletion Kit（New England Biolabs, Inc., Massachusetts, USA）according to the manufacturer's protocol. Secondly, sequencing libraries were prepared using NEBNext® Ultra™ II Directional RNA Library Prep Kit（New England Biolabs, Inc., Massachusetts, USA）. BioAnalyzer 2100 system（Agilent Technologies, USA）was used in quality control. The libraries were sequenced on an Illumina HiSeq 4000 platform (Illumina, San Diego, CA, USA) according to the manufacturer's instructions, and 150-bp paired-end reads were generated. Cutadapt (V1.9.3) [1] was used to acquire high-quality reads.

The high-quality reads were then mapped to the Homo sapiens Hg19 genome using STAR (V2.5.1b)[2]. CircRNAs were identified by DCC (V0.4.4)[3] and annotated by circBase[4] and Circ2Traits[5]. The differentially expressed circRNAs between HBx-oe and NC HepG2 cells were screened by edge R.[6] We used threshold values of ≥2 (or ≤0.5)-fold change and a P value <0.05.

Quantitative real-time PCR

Total RNAs were extracted using Trizol reagent (Invitrogen, CA). The first-strand cDNA was generated using the M-MLV Reverse Transcriptase kit (Invitrogen, CA) with random primers. Real-time PCR reactions were performed in the StepOne^{TM} Real-Time PCR System (Applied Biosystems, Foster City, CA). The real-time PCR reactions were performed in triplicate. ACTB were employed as endogenous control for mRNA and miRNAs, respectively. The relative expression was calculated using the comparative ΔΔCt method. The primer sequences are presented in Supplementary Table 6.

Transient transfection

The transient transfection of small interfering RNAs and plasmids were performed using the Lipofectamine 3000 kit (Invitrogen) according to the manufacturer's instructions. The siRNA sequences are listed in Supplementary Table 6.

Western blot analysis:

Total protein was extracted from snap-frozen tissues with RIPA Lysis Buffer and PMSF (Beyotime Co., China) according to the manufacturer's instructions. Western blotting was performed as described previously[7]. Antibody binding was detected with an Odyssey infrared scanner (Li-Cor Biosciences Inc.). The antibodies used are listed in Supplementary Table 7.

Cell lines

Hep3B, HepG2, Huh7 and MHCC97H cells were obtained from the Chinese Academy of Sciences Cell Bank and were authenticated by short tandem repeat (STR) profiling. Cells were grown in Dulbecco's modified Eagle's medium with 10% foetal bovine serum (Gibco BRL). Cells were maintained in an atmosphere of 5% CO_2 in a humidified 37°C incubator. Primary human hepatocyte (HH) (Lot #M00995-P) was obtained from RILDbiotech (Shanghai, China) and cultured as described previously[8].

RNA immunoprecipitation.

RNA immunoprecipitation (RIP) experiments were performed using a Magna RIP™ RNA-Binding Protein Immunoprecipitation Kit (Millipore, USA) according to the manufacturer's instructions.

Actinomycin D assay

The Actinomycin D assay was performed as previously described[9-11]. HCC cells were equally seeded in 5 wells in 24-well plates (5 × 104 cells per well). 24 hours later, the cells were exposed to actinomycin D (2μg/ml, Abcam, ab141058) for 0h, 12h, 24h and 48h, respectively. After that, the cells were harvested and the relative RNA levels of cFAM210A were analyzed by qRT-PCR and normalized to the values measured in the mock treatment group (the 0h group).

Promoter luciferase reporter assay

The promoter of RBM15 and MET were inserted into the vector GV238 (Genechem) respectively. Dual-luciferase reporter assay was carried out completely in line with the manufacturer's instructions (Beyotime, Shanghai, China).

Dual RNA fluorescence in situ hybridization (FISH) and immunofluorescence assay

CY3-labeled probe (Supplementary Table 5) to cFAM210A back-slice sequence was synthesized by RiboBio (Guangzhou, China). The probe signals were detected by the Fluorescent In Situ Hybridization Kit (RiboBio), following the manufacturer's instructions. HepG2 cells were incubated with antibodies specific for human YBX1 (20339-1-AP, Proteintech Group) at 4℃ overnight and then with FITC-labeled goat anti-rabbit IgG for 30 minutes. Subsequently, the nuclei were re-dyed with DAPI. Images were taken with Nikon Eclipse E200 Microscope (Japan).

Cell viability assays

Cell viability was evaluated using the cell counting kit 8 (CCK8; Dojindo, Kumamoto, Japan) assay. Transfected cells were plated onto a 96-well plate at a cell density of 2000 cells per well for 24 h. Next, the viability of the cells was measured at 450 nm using Synergy 2 (BioTek, USA) every 24 h for 4 days. 10 μL of CCK8 assay was added 2 hours before measurement.

5-Ethynyl-20-deoxyuridine (EdU) incorporation assays

The EdU assay was carried out with a Cell Light EdU DNA Cell Proliferation Kit (RiboBio, Shanghai, PR, China) according to the protocol as described before.[12] Images were acquired with Zeiss axiophot photomicroscope (Carl Zeiss) and Image-Pro plus 6.0 software, and the percentage of EdU-positive cells was calculated.

Sphere formation assays

The spheres formation assay was performed as previously described. [12] 1000 cells per well were seeded in ultra-low adherent-conditioned plates (Corning, USA) with serum-free DMEM medium containing B27 supplement (1:50; Invitrogen), 20 ng/mL epidermal growth factor (Invitrogen) and 20 ng/mL basic fibroblast growth factor (Invitrogen) for 14 days to test their ability of forming primary spheres. On day14, cell sphere number of spheres was counted using an inverted microscope (Olympus, Tokyo, Japan).

In vitro limiting dilution assays

HCC cells were seeded into 96-well ultra-low attachment culture dishes at cell doses described in the body of article and incubated in spheroid-forming conditions for 14 days. Sphere formation was assessed by visual inspection. Based on the frequency of wells without spheroids, the proportion of spheroid-initiating cells was determined using Poisson's distribution statistics and the L-Calcsoftware program (Version 1.1;

Stem Cell Technologies, Inc., Vancouver, British Columbia, Canada).

In vivo limiting dilution assays

Male nude mice were purchased from the Laboratory Animal Resources, Chinese Academy of Sciences (Beijing, China) and received humane care. Various amounts of HCC cells were injected subcutaneously of nude mice, as described before. [12] Kinetics of tumor formation were estimated twice weekly, and mice were monitored for 8 weeks. Frequency of tumor-initiating cells was determined using Poisson's distribution statistics and the L-Calc software program (Version 1.1; Stem Cell Technologies).

Supplementary Table 5. Clinical Characteristics of 20 liver hemangioma and 100 HCC patients in this study

	liver hemangioma	HCC	
	Cohort 1	Cohort 2	Cohort 3
All cases	20	20	80
Age, years,>50: ≤50	8:12	7:13	38:42
Gender, male/female	11:9	13:8	66:14
HBsAg, positive/negative	0:20	20:0	80:0
Liver cirrhosis, with/without	0:20	5:15	43:37
AFP, μg/L,>20: ≤20	0:20	16:4	54:26
Tumour size, cm,>5: ≤5	/	7:13	44:36
No. tumour, multiple: solitary	/	5:15	13:67
Edmondson’s grade, III+IV: I+II	/	16:4	64:16
Microvascular invasion, present: absent	/	4:16	25:55
Pathological satellite, present/absent	/	8:12	35:45
Encapsulation, incomplete/complete	/	13:7	47:33
TNM stage, II+III: I	/	4:16	19:61
BCLC stage, B+C: A	/	5:15	21:59

Supplementary Table 6. Primers, Probes and RNA sequences used in this study

**Primers for qRT-PCR**	
Primer Name	Sequence (5'-3')
ACTB-F	CCACCATGTACCCTGGCATTG
ACTB-R	TCATCTTGTTTTCTGCGCAAGTTA
ADAR1-F	CGAGAATCCCAAACAAGGAA
ADAR1-R	CTGGATTCCACAGGGATTGT
ALDH1A1-F	GCACGCCAGACTTACCTGTC
ALDH1A1-R	CCTCCTCAGTTGCAGGATTAAAG
ALKBH5-F	CCCGAGGGCTTCGTCAACA
ALKBH5-R	CGACACCCGAATAGGCTTGA
CBLL1-F	TGTGCAGCGAATTGAGCAGT
CBLL1-R	GCACGGGTAACAGGTTTTCCA
cBPTF-F	AAAGCTGACGGAATTTGTGGC
cBPTF-R	GTACCTGCATCTGGGGTGAC
CD133-F	AGTCGGAAACTGGCAGATAGC
CD133-R	GGTAGTGTTGTACTGGGCCAAT
CD13-F	GACCAAAGTAAAGCGTGGAATCG
CD13-R	TCTCAGCGTCACCCGGTAG
CD24-F	CTCCTACCCACGCAGATTTATTC
CD24-R	AGAGTGAGACCACGAAGAGAC
CD44-F	CTGCCGCTTTGCAGGTGTA
CD44-R	CATTGTGGGCAAGGTGCTATT
CD90-F	ATCGCTCTCCTGCTAACAGTC
CD90-R	CTCGTACTGGATGGGTGAACT
cDNA2-F	CACTTCGACTGACTTCCCTCA
cDNA2-R	CCAGGCGCTTTTCACAGTTT
cFAM210A-F	CACAGCTCAGGGAACTCCG
cFAM210A-R	TGCGTGCCAGTCGAGATAC
cFAM210A-MeRIP-F	AGCCTGATCCTTTGCAAGACA
cFAM210A-MeRIP-R	AGGGCCTTGTACCAAAACCA
cIQGAP1-F	AGAGAAAGAGATGTTTATGAGGAGC
cIQGAP1-R	AAGATGCCCCCAATCTTGCTA
cMGAT5-F	TTACCATCCAGCAGCGAACT
cMGAT5-R	TCTTTGCAAGCGGCCCAAAA
cPPP1R13B-F	CCAAGAGCAACGAACTCAGAGA
cPPP1R13B-R	CCTCCGTGGACCCCATTTC
cPRPSAP1-F	AGATCCTGAAAGAGAGAGGCG
cPRPSAP1-R	CGATGATTGCGATGCGGC
cTRIM24-F	CATACCGGTTACGGCACCTC

cTRIM24-R	CAGCTTCAGCTGCTCCTTTTT
cZCCHC2-F	GACTGAGATACGCACCTCCC
cZCCHC2-R	TGTGTACAGCTTCTCGCTGA
DHX9-F	TGCCTCCAAGAAAGTCCA
DHX9-R	TCCGCTTCCATTGTCGTAT
EPCAM-F	AATCGTCAATGCCAGTGTACTT
EPCAM-R	TCTCATCGCAGTCAGGATCATAA
FTO-F	TGGGTTCATCCTACAACGG
FTO-R	CCTCTTCAGGGCCTTCAC
FUS-F	AACTTCGTTGCTTGCTTGCC
FUS-R	TGGCCATAGCCTGAAGTGTC
HBx-F	CTGCAATGTCAACGACCGAC
HBx-R	TGCGCAGACCAATTTATGCC
HRSP12-F	GCTGCAAGAGGGAAGGCTTA
HRSP12-R	TCCACTTGAAGGGTCCATGC
KIAA1429-F	GAATACTGATGGTCTGGTGCTA
KIAA1429-R	CTTGGCTGTGGTCTTGGA
MDR1-F	GGGAGCTTAACACCCGACTTA
MDR1-R	GCCAAAATCACAAGGGTTAGCTT
MET-F	AGCAATGGGGAGTGTAAAGAGG
MET-R	CCCAGTCTTGTACTCAGCAAC
METTL14-F	AGAAACTTGCAGGGCTTCCT
METTL14-R	TCTTCTTCATATGGCAAATTTTCTT
METTL16-F	AGACCTCCGCCTAGTTCTGT
METTL16-R	GGGAACCCCTTGTATGCGAA
METTL3-F	AAGCTGCACTTCAGACGAAT
METTL3-R	GGAATCACCTCCGACACTC
mFAM210A-F	CCTTCGCCATAAGCAAGGGA
mFAM210A-R	CCACACTGTCAGGTAACCCA
NF90-F	GCCATTACGCCCATGAAACG
NF90-R	AATGAATTGCCATCAACCTCCA
POP1-F	AGCCATCTGATGAAGTGGGC
POP1-R	AAGTGGCCCAAGATGGACAG
QKI-F	CAAACGGAACTCCTCACCC
QKI-R	GCCACCGCACCTAATACAC
RBM15B-F	TACACGGAGGCTACCAGTACA
RBM15B-R	GTCGTACAGCCCGTAGTAGTC
RBM15-F	TGGTGTCCCTAAAGGGAGGAA
RBM15-R	AGGCCCATGTAAACTCCACA
WTAP-F	GCTTTGGAGGGCAAGTACAC
WTAP-F	TTGTAATGCGACTAGCAACCAA

WTAP-R	TCCTTGGTTGCTAGTCGCAT
WTAP-R	GCTGGGTCTACCATTGTTGATCT
YTHDF1-F	AATAACCAGCTCCGGCACAT
YTHDF1-R	AACTGGTTCGCCCTCATTGT
YTHDF2-F	TAGCCAGCTACAAGCACACC
YTHDF2-R	TGCAAGTCTGCAATCGTCTCT
ZC3H13-F	GTGCCGTAACTGGCTGAAGA
ZC3H13-R	CCTTTACCACGAGGTGAAGGG
**FISH probes**	
cFAM210A	5-CY3-GAUAGGUUUCAGCUUUUUCAAGGCUGCAUA-3
**siRNAs**	
cFAM210A-siRNA/shRNA	r(UGCAGCCUUGAAAAGCUGA)d(TT)
HRSP12-siRNA	r(UGUAAUAGGGAGAGUUGAA)d(TT)
POP1-siRNA	r(GAAUUUAACCGUAGACAAA)d(TT)
RBM15-siRNA	r(GGUGAUAGUUGGGCAUAUA)d(TT)
YTHDF2-siRNA	r(AAGGACGUUCCCAAUAGCCAA)d(TT)

Supplementary Table 7. The antibodies used in this study

Antibody	Supplier	Catalogue number	Primary/secondary	Host	Mono-/polyclonal
ACTB	Proteintech	66009-I-1g	P	mouse	m
ADAR1	Abcam	ab168809	P	rabbit	p
AGO2	Abcam	ab32381	P	rabbit	P
ALKBH5	Abcam	ab195377	P	rabbit	m
Anti-Flag	GenScript	A00187	P	mouse	m
CBLL1	Proteintech	21179-1-AP	P	rabbit	P
DHX9	Abcam	ab26271	P	rabbit	P
FAM210A	Abcam	ab151142	P	rabbit	P
FTO	Abcam	ab126605	P	rabbit	m
FUS	Abcam	ab124923	P	rabbit	m
GAPDH	Abcam	ab8245	P	mouse	m
HBx	Abcam	ab2741	P	mouse	m
Histone H3	Abcam	ab201456	P	rabbit	m
HRSP12	Proteintech	12930-1-AP	P	rabbit	P
KIAA1429	Abcam	ab271136	P	rabbit	m
MET	CST	8198	P	rabbit	m
METTL14	CST	51104	P	rabbit	m
METTL16	Proteintech	19924-1-AP	P	rabbit	P
METTL3	Affinity	DF12020	P	rabbit	P
NF90/NF110	Abcam	ab92355	P	rabbit	m
POP1	Proteintech	12029-1-AP	P	rabbit	P
P-AKT	Abcam	ab81283	P	rabbit	m
P-YBX1^{S102}	Abcam	ab138654	P	rabbit	P
QKI	Abcam	ab195960	P	rabbit	p
RBM15	Proteintech	10587-1-AP	P	rabbit	P
RBM15B	Proteintech	22249-1-AP	P	rabbit	P
RPS3	Proteintech	66046-1-Ig	P	mouse	m
SNRPF	Abcam	ab154870	P	rabbit	m
WTAP	Abcam	ab195380	P	rabbit	m
YBX1	Proteintech	20339-1-AP	P	rabbit	P
YTHDF2	Abcam	ab220163	P	rabbit	m
ZC3H13	Affinity	DF4623	P	rabbit	P
IRDye Goat anti-Mouse 680RD	LI-COR	926-68070	S	goat	p
IRDye Goat anti-Rabbit 800CW	LI-COR	926-32211	S	goat	p

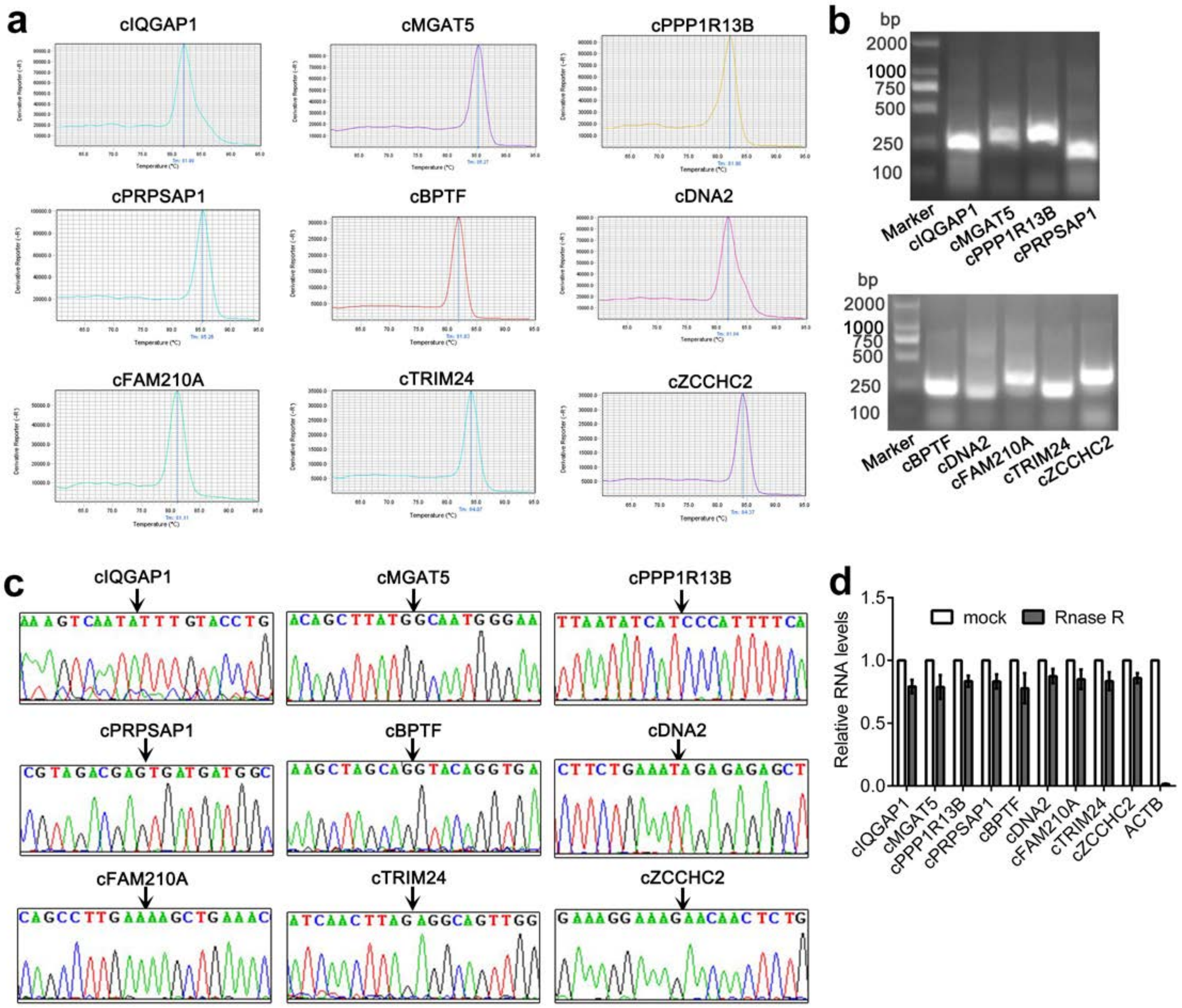

Supplementary Fig. 1. Validation of the structure and sizes of candidate circRNAs in HepG2. a Melting curves of qRT-PCR products of candicate circRNAs. **b** DNA electrophoresis showing the singularity of qRT-PCR products with divergent primers. **c** Sanger sequencing method showing back-sliced sites of candidates. **d** qRT-PCR after RNase R digestion verifying the circular structure of candidates, while ACTB mRNA were employed as a negative control.

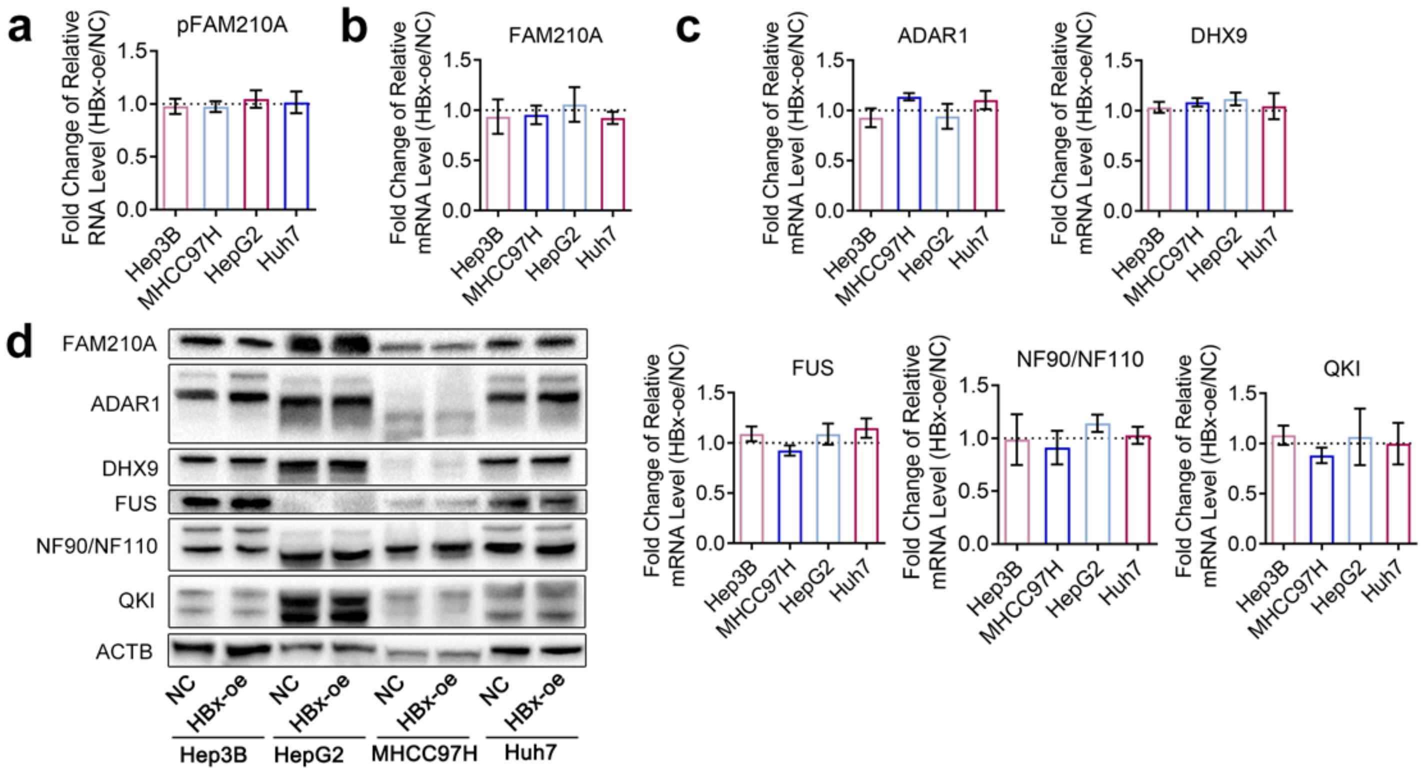

Supplementary Fig. 2. HBx could not affect the biogenesis of cFAM210A. a-c qRT-PCR showing the expression of pFAM210A, the mRNA of FAM210A, ADAR1, DHX9, FUS, NF90/NF110 and QKI in HBx-oe HCC cell lines (normalized to NC). **d** Western blotting showing the expression of FAM210A, ADAR1, DHX9, FUS, NF90/NF110 and QKI in HCC cell lines. ACTB was used as an endogenous control. pFAM210A, the precursor mRNA of FAM210A; NC, negative control. HBx-oe, HBx-overexpression.

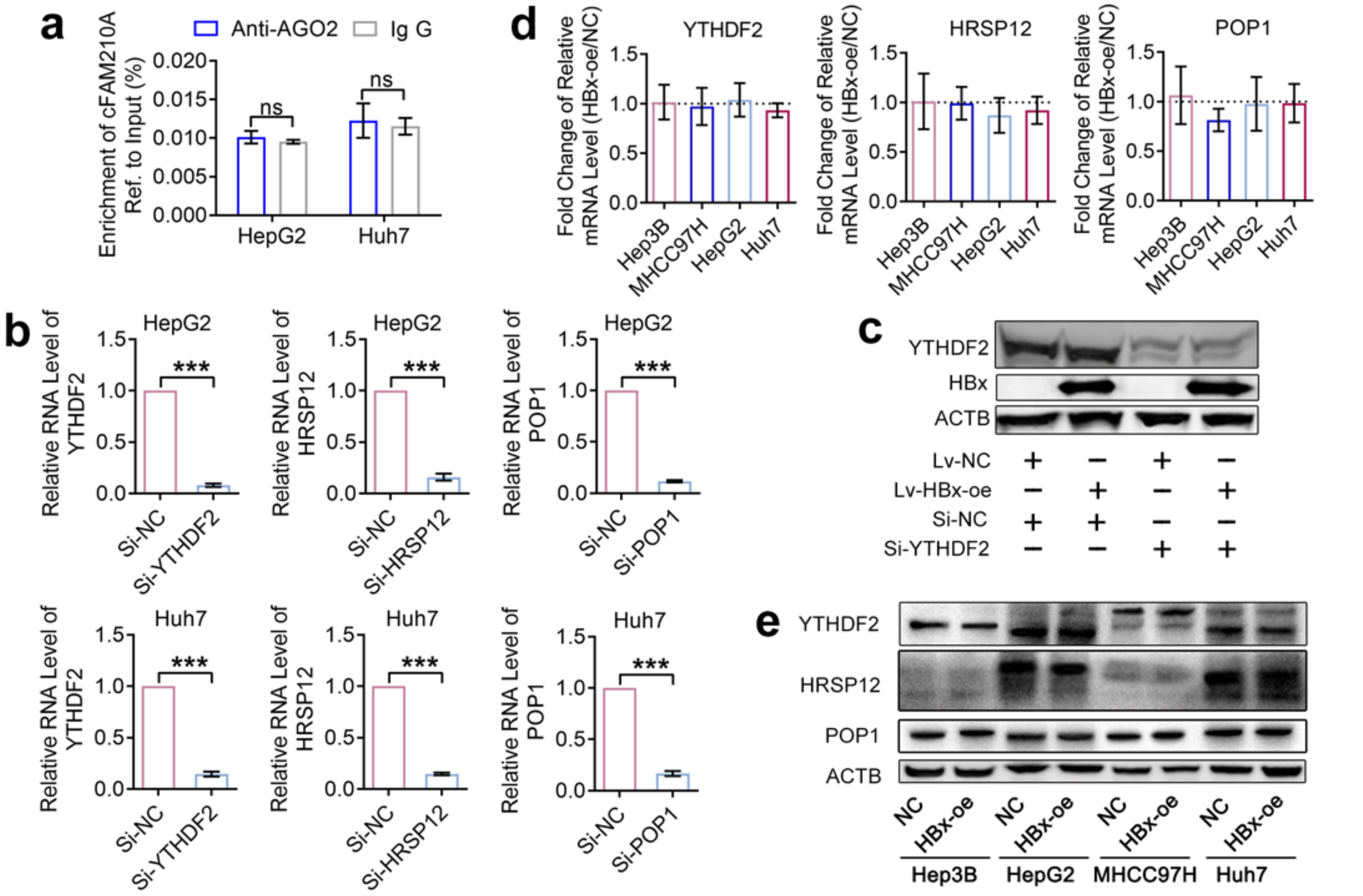

Supplementary Fig. 3. The experiments about whether HBx could affect the degradation of cFAM210A. a RIP-qPCR using anti-AGO2 antibody was performed in HCC cell lines. **b** qRT-PCR showing the expression of YTHDF2, HRSP12 and POP1 after using siRNAs. **c** Western blotting showing the expression of HBx and YTHDF2 in the rescue assay in HepG2 cells. **d** qRT-PCR showing the expression of YTHDF2, HRSP12 and POP1 in HBx-oe HCC cell lines (normalized to NC). **e** Western blotting showing the expression of YTHDF2, HRSP12 and POP1 in HCC cell lines. For (**b**) and (**d**), ACTB was used as an endogenous control. Student's t test was used. NC, negative control; HBx-oe, HBx-overexpression; ns, not significant. *, *P*<0.05; **, *P*<0.01; ***, *P*<0.001.

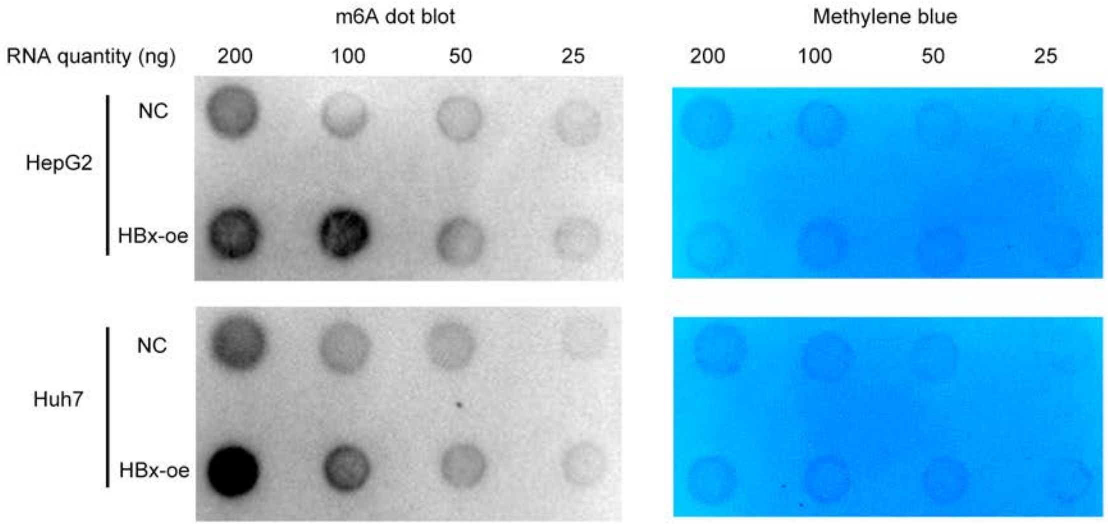

Supplementary Fig. 4. m6A dot blot assays in HCC cells. m6A dot blot assays showed the global m6A level of RNA extracted from HBx-overexpressing (HBx-oe) HCC cells and its negative controls. The intensity of dot immunoblotting indicated the m6A level of total RNAs, while methylene blue staining was applied to measure input RNA.

a Sequence of FAM210A protein (272AA, 31kDa):
MQWNVPRTVSRLARRTCLEPHNAGLFGHCQNVKGPLLLYNAESKVVLVQGP
QKQWLHLSAAQCVAKERRPLDAHPPQPGVLRHKQGKQHVSFRRVFSSSATAQ
GTPEKKEEPDPLQDKSISLYQRFKKTFRQYGKVLIPVHLITSGVWFGTFYYAAL
KGVNVVPFLELIGLPDSVVSILKNSQSGNALTAYALFKIATPARYTVTLGGTSV
TVKYLRSHGYMSTPPPVKEYLQDRMEETKELITEKMEETKDRLTEKLQETKE
KVSFKKKVE

Sequence of predicted cFAM210A protein (159AA, 18kDa):
MQWNVPRTVSRLARRTCLEPHNAGLFGHCQNVKGPLLLYNAESKVVLVQGP
QKQWLHLSAAQCVAKERRPLDAHPPQPGVLRHKQGKQHVSFRRVFSSSATAQ
GTPEKKEEPDPLQDKSISLYQRFKKTFRQYGKVLIPVHLITSGVWFGTFYYAAL
KS

Immunogen sequence of anti-FAM210A antibody (cat no. ab151142, Abcam):
QWNVPRTVSRLARRTCLEPHNAGLFGHCQNVKGPLLLYNAESKVVLVQGPQ
KQWLHLSAAQCVAKERRPLDAHPPQPGVLRHKQGKQHVSFRRVFSSSATAQG
TP

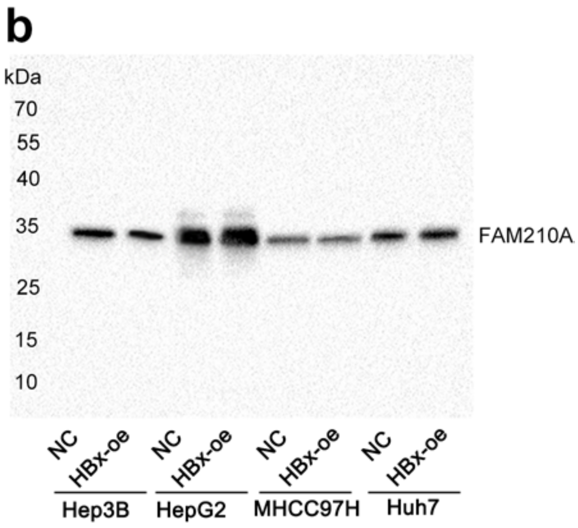

Supplementary Fig. 5. Western blotting showed that the predicted cFAM210A protein did not exist. a The sequences of FAM210A protein, predicted cFAM210A protein (predicted by circRNADB) and anti-FAM210A antibody (ab151142) immunogen. The overlapped sequences were highlighted. **b** Western blotting using anti-FAM210A antibody (ab151142) in HCC cells showed FAM210A protein, but not the predicted cFAM210A protein.

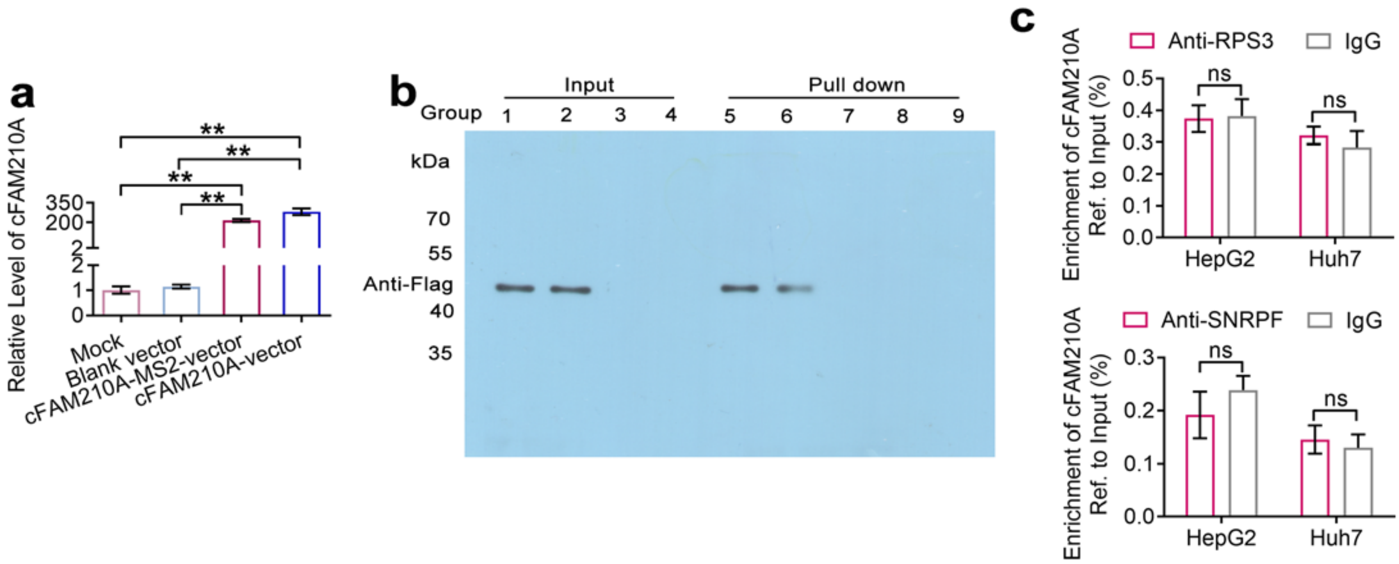

Supplementary Fig. 6. CircRNA pull down assay and its validation. a qRT-PCR showing the overexpressing effects of vectors in HepG2 cells. **b** Western blotting showed that the flagged proteins were successfully pulled down. **c** RIP-qPCR using anti-RPS3 and anti-SNRPF antibodies in HCC cell lines. For (**a**), ACTB was used as an endogenous control. For (**a**) and (**c**), Student's t test was used. ns, not significant. *, *P*<0.05; **, *P*<0.01; ***, *P*<0.001.

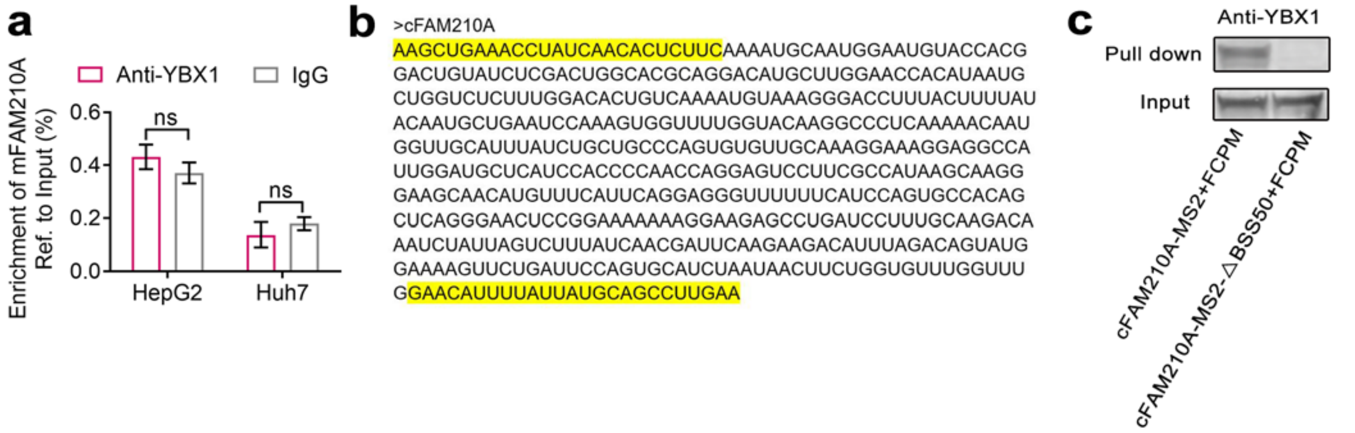

Supplementary Fig. 7. The determination of the key YBX1-binding sequence on cFAM210A. a RIP-qPCR using an anti-YBX1 antibody showed that mFAM210A can not bind to YBX1. **b** The sequence of cFAM210A. The 50 nucleotides across the its back-splicing site (BSS50) were highlighted. **c** Western blotting following circRNA pull down showed that YBX1 can be enriched by wild-type cFAM210A, but not cFAM210A with BSS50 deletion mutation (△BSS50). For (**a**), Student's t test was used. ns, not significant.

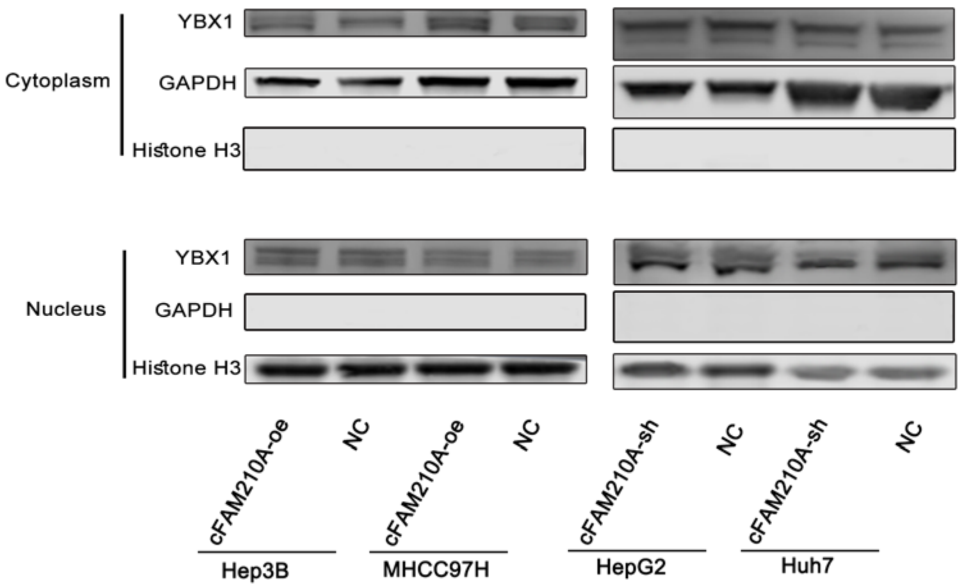

Supplementary Fig. 8. Nuclear extract and following Western blotting assays. Western blotting showing that the localization of YBX1 did not show significant changes after overexpressing or suppressing cFAM210A.

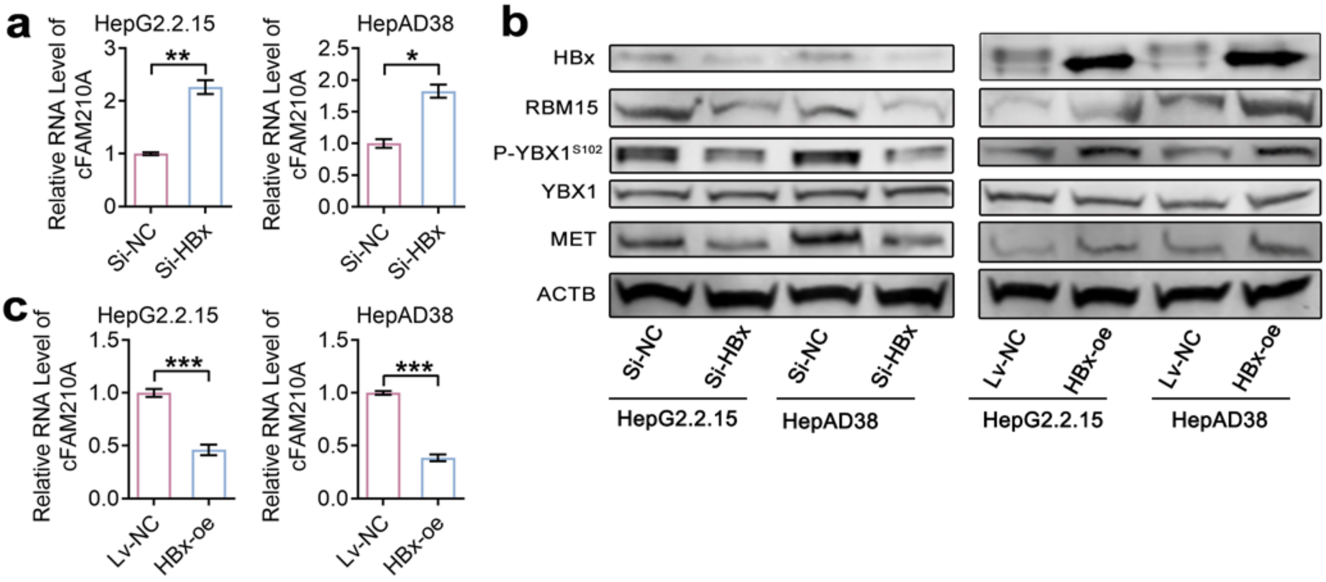

Supplementary Fig. 9. The role of HBx on the expression of cFAM210A, RBM15, P-YBX1^{S102}, YBX1 and MET in HBV-containing HepG2.2.15 and HepAD38 cells. a, c qPCR showing the expression of cFAM210A after silencing or overexpressing HBx. **b** Western blotting demonstrating the expression RBM15, P-YBX1^{S102}, YBX1 and MET after silencing or overexpressing HBx. For (**a**) and (**c**), Student's t test was used. NC, negative control; HBx-oe, HBx-overexpression. *, P<0.05; **, P<0.01; ***, P<0.001.

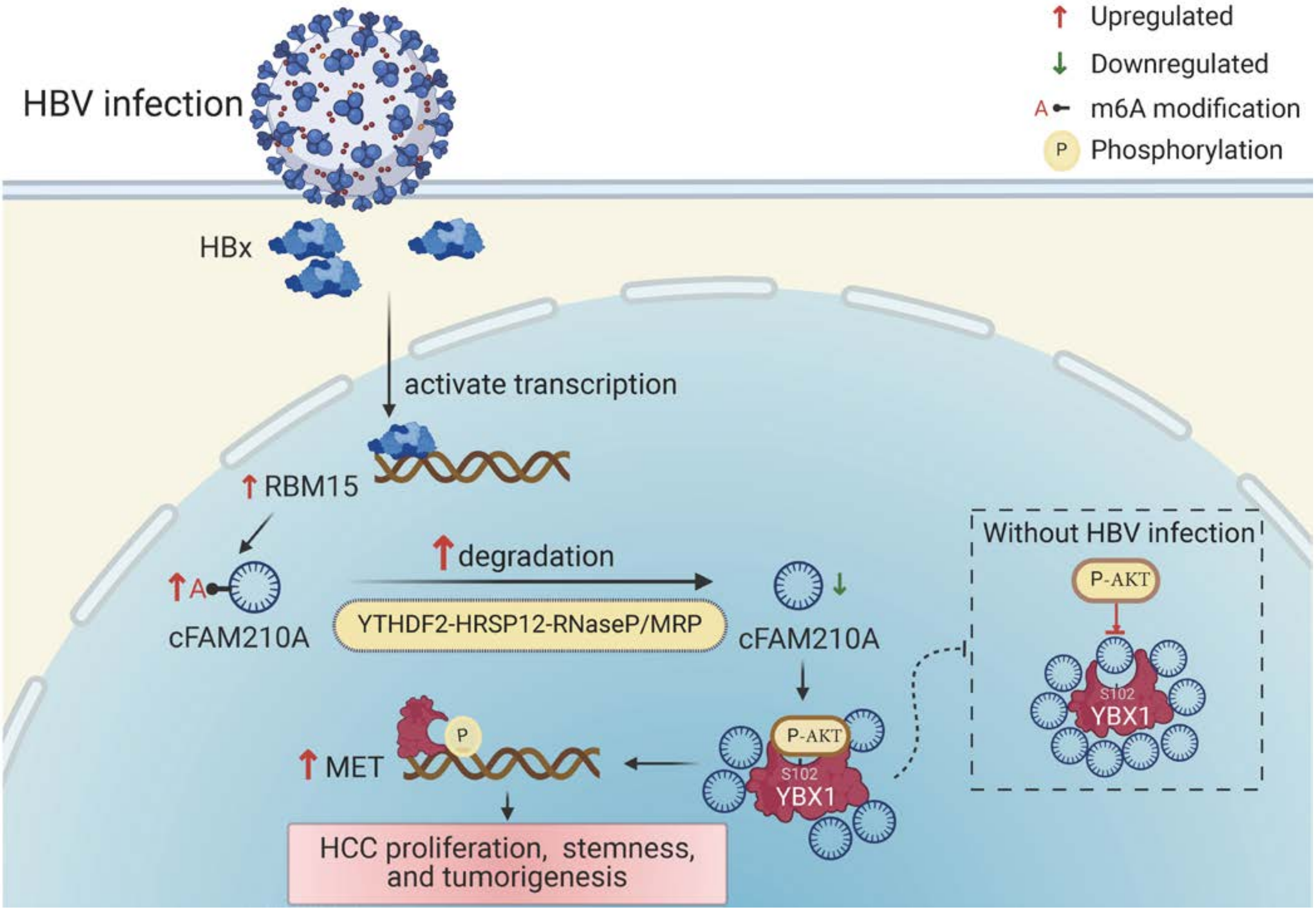

Supplementary Fig. 10. The graphical abstract of this study.

References

1 Kechin, A., Boyarskikh, U., Kel, A. & Filipenko, M. cutPrimers: A New Tool for Accurate Cutting of Primers from Reads of Targeted Next Generation Sequencing. *J Comput Biol* **24**, 1138-1143 (2017).

2 Dobin, A. *et al.* STAR: ultrafast universal RNA-seq aligner. *Bioinformatics* **29**, 15-21 (2013).

3 Cheng, J., Metge, F. & Dieterich, C. Specific identification and quantification of circular RNAs from sequencing data. *Bioinformatics* **32**, 1094-1096 (2016).

4 Glažar, P., Papavasileiou, P. & Rajewsky, N. circBase: a database for circular RNAs. *Rna* **20**, 1666-1670 (2014).

5 Ghosal, S., Das, S., Sen, R., Basak, P. & Chakrabarti, J. Circ2Traits: a comprehensive database for circular RNA potentially associated with disease and traits. *Front Genet* **4**, 283 (2013).

6 Robinson, M. D., McCarthy, D. J. & Smyth, G. K. edgeR: a Bioconductor package for differential expression analysis of digital gene expression data. *Bioinformatics* **26**, 139-140 (2010).

7 Anderson, S. F., Schlegel, B. P., Nakajima, T., Wolpin, E. S. & Parvin, J. D. BRCA1 protein is linked to the RNA polymerase II holoenzyme complex via RNA helicase A. *Nat Genet* **19**, 254-256 (1998).

8 Chen, Y. *et al.* Assessment of long-term functional maintenance of primary human hepatocytes to predict drug-induced hepatoxicity in vitro. *Arch Toxicol* **95**, 2431-2442 (2021).

9 Kleeff, J., Kornmann, M., Sawhney, H. & Korc, M. Actinomycin D induces apoptosis and inhibits growth of pancreatic cancer cells. *Int J Cancer* **86**, 399-407 (2000).

10 Li, F. *et al.* Circular RNA ITCH has inhibitory effect on ESCC by suppressing the Wnt/beta-catenin pathway. *Oncotarget* **6**, 6001-6013 (2015).

11 Zheng, Q. *et al.* Circular RNA profiling reveals an abundant circHIPK3 that regulates cell growth by sponging multiple miRNAs. *Nat Commun* **7**, 11215 (2016).

12 Ding, W. B. *et al.* HBV/Pregenomic RNA Increases the Stemness and Promotes the Development of HBV-Related HCC Through Reciprocal Regulation With Insulin-Like Growth Factor 2 mRNA-Binding Protein 3. *Hepatology* **74**, 1480-1495 (2021).
